# The clinical landscape of CAR NK cells

**DOI:** 10.1186/s40164-025-00633-8

**Published:** 2025-03-27

**Authors:** Lasse Vedel Jørgensen, Emil Birch Christensen, Mike Bogetofte Barnkob, Torben Barington

**Affiliations:** 1 Department of Clinical Immunology, Odense University Hospital, Odense, Denmark; 2 Centre for Cellular Immunotherapy of Haematological Cancer Odense (CITCO), Odense, Denmark

**Keywords:** Chimeric antigen receptor, Natural killer cells, Clinical trials, Immunotherapy, Cancer, Autoimmune diseases

## Abstract

Chimeric antigen receptor (CAR) NK cell therapy has emerged as a promising alternative to CAR T cell therapy, offering significant advantages in terms of safety and versatility. Here we explore the current clinical landscape of CAR NK cells, and their application in hematologic malignancies and solid cancers, as well as their potential for treating autoimmune disorders. Our analysis draws from data collected from 120 clinical trials focused on CAR NK cells, and presents insights into the demographics and characteristics of these studies. We further outline the specific targets and diseases under investigation, along with the major cell sources, genetic modifications, combination strategies, preconditioning- and dosing regimens, and manufacturing strategies being utilized. Initial results from 16 of these clinical trials demonstrate promising efficacy of CAR NK cells, particularly in B cell malignancies, where response rates are comparable to those seen with CAR T cells but with lower rates of severe adverse effects, such as cytokine release syndrome (CRS), immune effector cell-associated neurotoxicity syndrome (ICANS), and graft-versus-host disease (GvHD). However, challenges remain in solid tumor applications, where only modest efficacy has been observed to date. Our analysis reveals that research is increasingly focused on enhancing CAR NK cell persistence, broadening their therapeutic targets, and refining manufacturing processes to improve accessibility and scalability. With recent advancements in NK cell engineering and their increased clinical applications, CAR NK cells are predicted to become an integral component of next-generation immunotherapies, not only for cancer but potentially for immune-mediated diseases as well.

**Supplementary Information:**

The online version contains supplementary material available at 10.1186/s40164-025-00633-8.

## Introduction

In recent decades cellular immunotherapies have emerged as a promising new tool in our repertoire of treatments against life-threatening diseases, including cancer. Its recent success is partly owed to the advent of genetic engineering of adoptively transferred immune cells to enhance their efficacy. Especially chimeric antigen receptor (CAR) T cell therapy has revolutionized the field, by combining the inherent anti-tumor activity of T cells with tumor targeting synthetic receptors [1]. CAR T cell therapy has shown remarkable efficacies in clinical trials against certain cancers, and so far seven CAR T cell products have been approved by the U.S. Food and Drug Administration (FDA), and six by the European Medicines Agency (EMA) for the treatment of hematologic malignancies [2]. Despite these advances, CAR T cell therapy faces major limitations and challenges that hinder its widespread adoption. First, autologous CAR T cell production is costly and complex, requiring patient leukapheresis, lengthy manufacturing, and logistics, which prolong vein-to-vein times [3]. This is particularly problematic for critically ill patients that may progress while waiting for the treatment. Additionally, previous treatments can impair the quality of leukapheresis products, leading to manufacturing failures or poor quality of the CAR T product [4]. High production costs and limited scalability further burden autologous CAR T cells, while allogeneic CAR T cells require extensive gene editing to prevent graft-versus-host disease (GvHD) and rejection [5, 6]. These limitations have spurred interest in alternative immune cells for CAR-based therapies, with NK cells emerging as promising candidates to overcome the challenges faced by T cells.

NK cells are large granular lymphocytes integral to the innate immune response against infected and transformed cells [7]. Unlike T and B cells, which rely on a single somatically rearranged receptor, NK cells use a variety of germline-encoded activating and inhibitory receptors, balancing these signals to trigger or inhibit functions like cytotoxic granule release and cytokine secretion [8]. This receptor diversity enables NK cells to eliminate target cells without prior activation [9, 10]. These attributes have made NK cells especially attractive in clinical contexts. Like T cells, NK cells can be modified with a CAR, which directs their specificity towards the malignant cells. However, CAR NK cells offer unique advantages over CAR T cells. For instance, they may be less sensitive to tumor escape caused by CAR antigen loss, as they retain CAR-independent cytotoxicity [11]. Additionally, CAR NK cells exhibit a distinct safety profile, characterized by a significantly reduced risk of cytokine release syndrome (CRS). This reduced risk is attributed to the intrinsic biology of NK cells, which secrete lower levels of pro-inflammatory cytokines such as interleukin-1β (IL-1β), IL-6, and IL-10 upon target cell engagement [12, 13]. Furthermore, NK cells do not cause GvHD when transplanted across human leukocyte antigen (HLA) barriers, making them suitable for allogeneic “off-the-shelf” therapies. Such therapies could replace autologous CAR T cells and enable the production of readily available, scalable doses [14].

However, clinical research on CAR NK cell therapy is still in its infancy with limited data on their safety and effectiveness compared to CAR T cells. Further, the role of manufacturing strategy and starting material used to produce CAR NK cells, is still under investigation. This review provides a comprehensive update on the clinical landscape of CAR NK cells, covering disease targets, manufacturing, treatment protocols, and early efficacy and safety results.

## Overview of clinical trials with CAR NK cells

Over a thousand clinical trials exploring CAR T cell therapies are currently registered on clinicaltrials.gov with their characteristics thoroughly reviewed in existing literature [15, 16]. In contrast, CAR NK cell therapy trials are relatively fewer, and a comprehensive summary of such trials is not yet available. To address this gap, we collected data from multiple clinical trial databases, including clinicaltrials.gov, chictr.org.cn, rctportal.niph.go.jp, as well as corporate databases. This effort identified 124 registered CAR NK cell therapy trials, detailed in Tables 1, 2, 3 and Supplementary Table 1, excluding four trials that were suspended, withdrawn, or terminated without patient enrollment. The data cut-off date was the 25th of October, 2024.

**Table 1:** Clinical trials of CAR NK cell agents for solid cancers

Target	Disease	Cell source	Phase	First posted	Current status	Enrollment	Sponsors	Identifier
5T4	Solid Tumors, unspecified	Undisclosed	Early Phase 1	2021-11-24	Recruiting	56	Shanghai East Hospital	NCT05137275
5T4	Solid Tumors, unspecified	Undisclosed	Early Phase 1	2021-12-30	Recruiting	40	Wuxi People’s Hospital	NCT05194709
AXL, CLDN6,GPC3,Mesothelin	Ovarian Cancer, Testis Cancer, Endometrial Cancer	Peripheral Blood	Phase 1	2022-06-01	Recruiting	200	Second Affiliated Hospital of Guangzhou Medical University	NCT05410717
CD70	Renal Cell Carcinoma, Mesothelioma, Osteosarcoma	Cord Blood	Phase 1/2	2023-03-29	Recruiting	50	M.D. Anderson Cancer Center	NCT05703854
Claudin18.2	Pancreatic Cancer, Gastric Cancer	Cord Blood	Phase 1	2024-07-19	Recruiting	30	Zhejiang Provincial People’s Hospital	NCT06464965
DLL3	Small Cell Lung Cancer	Undisclosed	Phase 1	2022-09-01	Recruiting	18	Tianjin Medical University Cancer Institute and Hospital	NCT05507593
GPC3	Ovarian Cancer	iPSC	Phase 1	2021-03-18	Recruiting	18	National Cancer Center Hospital East	jRCT2033200431
GPC3	Hepatocellular Carcinoma	Undisclosed	Undisclosed	2024-11-30	Not yet recruiting	12	Shanghai General Hospital, Shanghai Jiao Tong University School of Medicine	NCT06652243
HER2	Glioblastoma	NK-92	Phase 1	2017-12-01	Active, not recruiting	42	Johann Wolfgang Goethe University Hospital	NCT03383978
HER2	Breast Cancer, Gastric Cancer, Gastroesophageal Junction Adenocarcinoma	Undisclosed	Phase 1/2	2023-01-01	Not yet recruiting	133	Artiva Biotherapeutics	NCT05678205
Mesothelin	Ovarian Cancer	Peripheral Blood	Early Phase 1	2019-03-01	Unknown	30	Allife Medical Science and Technology Co., Ltd	NCT03692637
Mesothelin	Ovarian Cancer	Undisclosed	Phase 1	2021-06-30	Not yet recruiting	5	Zhuhai People’s Hospital	ChiCTR2100048100
MICA/B	Non-Small Cell Lung Cancer, Colorectal Cancer, Breast Cancer, Ovarian Cancer, Pancreatic Cancer, Head and Neck Squamous Cell Carcinoma, Gastroesophageal Junction Adenocarcinoma	iPSC	Phase 1	2022-05-31	W/T/S	5	Fate Therapeutics	NCT05395052
MUC1	Hepatocellular Carcinoma, Non-Small Cell Lung Cancer, Pancreatic Cancer, Triple-Negative Breast Cancer, Glioblastoma, Colorectal Cancer, Gastric Cancer	NK-92	Phase 1/2	2016-07-01	Unknown	10	PersonGen BioTherapeutics (Suzhou) Co., Ltd	NCT02839954
NKG2DL	Solid Tumors, unspecified	Peripheral Blood	Phase 1	2018-01-02	Unknown	30	The Third Affiliated Hospital of Guangzhou Medical University	NCT03415100
NKG2DL	Colorectal Cancer	Undisclosed	Phase 1	2021-12-10	Recruiting	38	Zhejiang University	NCT05213195
NKG2DL	Solid Tumors, unspecified	NK-92	Phase 1	2023-01-26	Recruiting	20	Xinxiang medical university	NCT05528341
NKG2DL	Ovarian Cancer	Undisclosed	Undisclosed	2023-03-01	Recruiting	18	Hangzhou Cheetah Cell Therapeutics Co., Ltd	NCT05776355
NKG2DL	Pancreatic Cancer	Undisclosed	Early Phase 1	2024-06-30	Not yet recruiting	20	Zhejiang University	NCT06478459
NKG2DL	Pancreatic Cancer	Undisclosed	Early Phase 1	2024-07-31	Not yet recruiting	30	Zhejiang University	NCT06503497
PD-L1	Non-Small Cell Lung Cancer	NK-92	Phase 1	2018-09-29	Completed	2	Xinxiang medical university	NCT03656705
PD-L1	Non-Small Cell Lung Cancer, Small Cell Lung Cancer, Urothelial Carcinoma, Head and Neck Squamous Cell Carcinoma, Merkel Cell Carcinoma, Melanoma, Renal Cell Carcinoma, Gastric Cancer, Cervical Cancer, Hepatocellular Carcinoma, Colorectal Cancer	NK-92	Phase 2	2018-12-11	Active, not recruiting	147	ImmunityBio, Inc	NCT03228667
PD-L1	Solid Tumors, unspecified	NK-92	Phase 1	2019-07-18	Active, not recruiting	16	ImmunityBio, Inc	NCT04050709
PD-L1	Pancreatic Cancer	NK-92	Phase 2	2020-07-21	Active, not recruiting	328	ImmunityBio, Inc	NCT04390399
PD-L1	Triple-Negative Breast Cancer	NK-92	Phase 1/2	2021-09-27	W/T/S	3	ImmunityBio, Inc	NCT04927884
PD-L1	Gastric Cancer, Gastroesophageal Junction Adenocarcinoma, Head and Neck Squamous Cell Carcinoma	NK-92	Phase 2	2021-12-14	Recruiting	55	National Cancer Institute (NCI)	NCT04847466
PD-L1	Glioblastoma	NK-92	Phase 2	2023-09-28	Recruiting	20	ImmunityBio, Inc	NCT06061809
PD-L1	HNSCC	NK-92	Phase 2	2023-12-07	Not yet recruiting	40	National Cancer Institute (NCI)	NCT06161545
PD-L1	HNSCC	NK-92	Phase 2	2024-02-16	Recruiting	25	ImmunityBio, Inc	NCT06239220
PSMA	Metastatic Castration-Resistant Prostate Cancer	iPSC	Early Phase 1	2018-12-01	Recruiting	9	Allife Medical Science and Technology Co., Ltd	NCT03692663
ROBO1	Solid Tumors, unspecified	NK-92	Phase 1/2	2019-05-01	Unknown	20	Asclepius Technology Company Group (Suzhou) Co., Ltd	NCT03940820
ROBO1	Solid Tumors, unspecified	NK-92	Phase 1/2	2019-05-01	Unknown	20	Asclepius Technology Company Group (Suzhou) Co., Ltd	NCT03931720
ROBO1	Pancreatic Cancer	NK-92	Phase 1/2	2019-05-01	Unknown	9	Asclepius Technology Company Group (Suzhou) Co., Ltd	NCT03941457
TROP2	NSCLC, Breast Cancer	Cord Blood	Phase 1	2023-10-04	Recruiting	54	M.D. Anderson Cancer Center	NCT06066424
TROP2	Pancreatic Cancer, Ovarian Cancer, Adenocarcinoma	Cord Blood	Phase 1/2	2023-12-31	Recruiting	51	M.D. Anderson Cancer Center	NCT05922930
TROP2	Colorectal Cancer	Cord Blood	Phase 1	2024-04-10	Not yet recruiting	42	M.D. Anderson Cancer Center	NCT06358430
TROP2	Non-Small Cell Lung Cancer	Undisclosed	Phase 1/2	2024-06-06	Not yet recruiting	50	Henan Cancer Hospital	NCT06454890
Undisclosed	Triple-Negative Breast Cancer	Undisclosed	Early Phase 1	2023-02-01	Not yet recruiting	12	First Affiliated Hospital of Shantou University Medical College	NCT05686720
Undisclosed	Hepatocellular Carcinoma	Undisclosed	Undisclosed	2023-05-04	Recruiting	12	Shantou University Medical College	NCT05845502
Undisclosed	Ovarian Cancer	Undisclosed	Early Phase 1	2023-06-1	Recruiting	12	Shantou University Medical College	NCT05856643
Undisclosed	Solid Tumors, unspecified	Undisclosed	Early Phase 1	2024-03-21	Active, not recruiting	10	The Second Hospital of Shandong University	NCT06572956

**Table 2:** Clinical trials of CAR NK cell agents for hematologic cancers

Target	Disease	Cell source	Phase	First posted	Current status	Enrollment	Sponsors	Identifier
BCMA	Multiple Myeloma	NK-92	Phase 1/2	2019-05-01	Unknown	20	Asclepius Technology Company Group (Suzhou) Co., Ltd	NCT03940833
BCMA	Multiple Myeloma	Cord Blood	Early Phase 1	2021-10-01	Recruiting	27	Xinqiao Hospital of Chongqing	NCT05008536
BCMA	Multiple Myeloma	iPSC	Phase 1	2021-11-24	Active, not recruiting	31	Fate Therapeutics	NCT05182073
BCMA	Multiple Myeloma	Undisclosed	Phase 1	2022-09-01	Not yet recruiting	34	Legend Biotech	NCT05498545
BCMA	Multiple Myeloma	Undisclosed	Early Phase 1	2022-11-13	Recruiting	19	Shenzhen Pregene Biopharma Co., Ltd	NCT05652530
BCMA	Multiple Myeloma, Plasma Cell Leukemia	Undisclosed	Early Phase 1	2023-09-21	Recruiting	18	Hrain Biotechnology Co., Ltd	NCT06045091
BCMA	Multiple Myeloma	Undisclosed	Phase 1	2024-08-30	Not yet recruiting	10	Shahid Beheshti University of Medical Sciences	NCT06242249
BCMA/GPRC5D	Multiple Myeloma	Undisclosed	Undisclosed	2024-09-10	Not yet recruiting	18	RenJi Hospital	NCT06594211
CD123	Acute Myeloid Leukemia	Undisclosed	Early Phase 1	2022-10-01	Recruiting	12	Affiliated Hospital to Academy of Military Medical Sciences	NCT05574608
CD123	Acute Myeloid Leukemia, Blastic Plasmacytoid Dendritic Cell Neoplasm (BPDCN)	Undisclosed	Phase 1/2	2023-08-31	Recruiting	36	Chongqing Precision Biotech Co., Ltd	NCT06006403
CD123	Acute Myeloid Leukemia	Undisclosed	Early Phase 1	2023-12-30	Recruiting	12	Peking University People’s Hospital	NCT06201247
CD19	Acute Lymphoblastic Leukemia	Peripheral Blood	Phase 1	2009-10-01	Completed	14	St. Jude Children’s Research Hospital	NCT00995137
CD19	Acute Lymphoblastic Leukemia	Peripheral Blood	Phase 1	2013-09-01	W/T/S	20	National University Health System, Singapore	NCT01974479
CD19	Acute Lymphoblastic Leukemia, Chronic Lymphocytic Leukemia, Follicular Lymphoma, Mantle Cell Lymphoma, Diffuse Large B-Cell Lymphoma	NK-92	Phase 1/2	2016-09-01	Unknown	10	PersonGen BioTherapeutics (Suzhou) Co., Ltd	NCT02892695
CD19	Acute Lymphoblastic Leukemia, Chronic Lymphocytic Leukemia, Non-Hodgkin’s Lymphoma, unspecified	Cord Blood	Phase 1/2	2017-06-21	Completed	49	M.D. Anderson Cancer Center	NCT03056339
CD19	B-Cell Lymphoma, unspecified	iPSC	Early Phase 1	2019-02-01	Unknown	10	Allife Medical Science and Technology Co., Ltd	NCT03824951
CD19	B-Cell Lymphoma, unspecified	iPSC	Early Phase 1	2019-03-01	Unknown	9	Allife Medical Science and Technology Co., Ltd	NCT03690310
CD19	Diffuse Large B-Cell Lymphoma	NK-92	Phase 1	2019-09-16	W/T/S	0	ImmunityBio, Inc	NCT04052061
CD19	Mantle Cell Lymphoma, Diffuse Large B-Cell Lymphoma, Follicular Lymphoma	Cord Blood	Phase 1/2	2019-10-03	W/T/S	0	M.D. Anderson Cancer Center	NCT03579927
CD19	B-Cell Lymphoma, unspecified, Chronic Lymphocytic Leukemia	iPSC	Phase 1	2020-03-19	W/T/S	98	Fate Therapeutics	NCT04245722
CD19	Non-Hodgkin’s Lymphoma, unspecified	Undisclosed	Early Phase 1	2020-12-17	Not yet recruiting	9	Xinqiao Hospital of Chongqing	NCT04639739
CD19	Acute Lymphoblastic Leukemia, Chronic Lymphocytic Leukemia, Non-Hodgkin’s Lymphoma, unspecified	Cord Blood	Phase 1	2021-04-10	Recruiting	27	Wuhan Union Hospital, China	NCT04796675
CD19	Non-Hodgkin’s Lymphoma, unspecified	Peripheral Blood	Phase 1	2021-05-01	Recruiting	25	Second Affiliated Hospital, School of Medicine, Zhejiang University	NCT04887012
CD19	Acute Lymphoblastic Leukemia, Diffuse Large B-Cell Lymphoma, Mantle Cell Lymphoma, Indolent Lymphoma, Waldenstrom Macroglobulinemia, Chronic Lymphocytic Leukemia, Small Lymphocytic Lymphoma	Peripheral Blood	Phase 1	2021-08-20	Recruiting	150	Nkarta Inc	NCT05020678
CD19	B-Cell Lymphoma, unspecified, Acute Lymphoblastic Leukemia	iPSC	Phase 1	2021-11-04	Recruiting	24	Zhejiang University	NCT05379647
CD19	Non-Hodgkin’s Lymphoma, unspecified	Cord Blood	Phase 2	2021-11-22	Active, not recruiting	27	Takeda	NCT05020015
CD19	Acute Lymphoblastic Leukemia, Chronic Lymphocytic Leukemia, Non-Hodgkin’s Lymphoma, unspecified	Undisclosed	Phase 1	2022-05-25	Recruiting	15	Beijing Boren Hospital	NCT05410041
CD19	Acute Lymphoblastic Leukemia	Undisclosed	Phase 1	2022-07-21	Completed	2	Shanghai Simnova Biotechnology Co.,Ltd	NCT05563545
CD19	Non-Hodgkin’s Lymphoma, unspecified	Cord Blood	Phase 1	2022-09-10	Recruiting	48	Second Affiliated Hospital, School of Medicine, Zhejiang University	NCT05472558
CD19	B-Cell Lymphoma, unspecified, B-Cell Leukemia, unspecified	Undisclosed	Phase 1/2	2022-10-01	W/T/S	0	Kunming Hope of Health Hospital	NCT05570188
CD19	B-Cell Lymphoma, unspecified, B-Cell Leukemia, unspecified	Undisclosed	Phase 1	2022-12-01	Recruiting	12	Affiliated Hospital to Academy of Military Medical Sciences	NCT05645601
CD19	Diffuse Large B-Cell Lymphoma, Indolent Lymphoma, Follicular Lymphoma, Mantle Cell Lymphoma, Marginal Zone Lymphoma	iPSC	Phase 1	2022-12-01	W/T/S	0	Fate Therapeutics	NCT05934097
CD19	B-Cell Lymphoma, unspecified, B-Cell Leukemia, unspecified	Undisclosed	Phase 1/2	2022-12-08	Recruiting	30	920th Hospital of Joint Logistics Support Force of People’s Liberation Army of China	NCT05654038
CD19	Non-Hodgkin’s Lymphoma, unspecified	iPSC	Phase 1	2023-01-24	Recruiting	75	Century Therapeutics, Inc	NCT05336409
CD19	Diffuse Large B-Cell Lymphoma	Undisclosed	Early Phase 1	2023-03-01	Recruiting	12	Changhai Hospital	NCT05673447
CD19	Acute Lymphoblastic Leukemia, B-Cell Lymphoma, unspecified, Chronic Lymphocytic Leukemia	Peripheral Blood	Early Phase 1	2023-03-01	Recruiting	12	Xuzhou Medical University	NCT05739227
CD19	Non-Hodgkin’s Lymphoma, unspecified	NK-92	Phase 1	2023-09-01	Recruiting	20	ImmunityBio, Inc	NCT05618925
CD19	B-Cell Lymphoma, unspecified, B-Cell Leukemia, unspecified	iPSC	Phase 1	2023-09-01	Recruiting	166	Fate Therapeutics	NCT05950334
CD19	B-Cell Lymphoma, unspecified	Undisclosed	Phase 1	2024-01-04	Recruiting	55	Shanghai Simnova Biotechnology Co.,Ltd	NCT06206902
CD19	Non-Hodgkin’s Lymphoma, unspecified	NK-92	Phase 1	2024-05-01	Not yet recruiting	10	ImmunityBio, Inc	NCT06334991
CD19	Diffuse Large B-Cell Lymphoma, Mantle Cell Lymphoma, Primary Mediastinal B-cell Lymphoma	Cord Blood	Phase 1	2024-06-10	Not yet recruiting	52	Second Affiliated Hospital, School of Medicine, Zhejiang University	NCT06464861
CD19	Acute Lymphoblastic Leukemia	Undisclosed	Phase 1/2	2024-12-19	Not yet recruiting	10	Shahid Beheshti University of Medical Sciences	NCT06631040
CD19/CD22	B-Cell Lymphoma, unspecified	iPSC	Early Phase 1	2019-02-01	Unknown	10	Allife Medical Science and Technology Co., Ltd	NCT03824964
CD19/CD70	Non-Hodgkin’s Lymphoma, unspecified	Cord Blood	Phase 1	2022-12-15	Recruiting	48	Second Affiliated Hospital, School of Medicine, Zhejiang University	NCT05667155
CD19/CD70	Non-Hodgkin’s Lymphoma, unspecified	Cord Blood	Phase 1/2	2023-01-18	Recruiting	48	Aibin Liang	NCT05842707
CD20	B-Cell Lymphoma, unspecified	Peripheral Blood	Phase 1	2024-10-01	Recruiting	30	Interius BioTherapeutics Inc	NCT06539338
CD22	B-Cell Lymphoma, unspecified	iPSC	Early Phase 1	2019-03-01	Unknown	9	Allife Medical Science and Technology Co., Ltd	NCT03692767
CD33	Acute Myeloid Leukemia	NK-92	Phase 1/2	2016-10-01	Unknown	10	PersonGen BioTherapeutics (Suzhou) Co., Ltd	NCT02944162
CD33	Acute Myeloid Leukemia	Undisclosed	Phase 1	2021-12-23	Recruiting	27	Xinqiao Hospital of Chongqing	NCT05008575
CD33	Acute Myeloid Leukemia	Undisclosed	Phase 1	2022-10-28	Recruiting	18	Institute of Hematology & Blood Diseases Hospital	NCT05601466
CD33	Acute Myeloid Leukemia	Undisclosed	Phase 1	2022-12-24	Recruiting	19	Zhejiang University	NCT05665075
CD33, CLL1	Acute Myeloid Leukemia	iPSC	Phase 1	2024-04-16	Recruiting	24	Zhejiang University	NCT06367673
CD33/CLL1	Acute Myeloid Leukemia	Undisclosed	Early Phase 1	2020-11-30	Recruiting	18	Wuxi People’s Hospital	NCT05215015
CD33/CLL1	Acute Myeloid Leukemia	Undisclosed	Phase 1	2021-06-07	Recruiting	27	The Affiliated Hospital of Xuzhou Medical University	ChiCTR2100047084
CD33/CLL1	Acute Myeloid Leukemia	iPSC	Phase 1	2023-08-10	Not yet recruiting	102	Institute of Hematology & Blood Diseases Hospital	NCT05987696
CD33/FLT3	Acute Myeloid Leukemia, Myelodysplastic Syndromes	Peripheral Blood	Phase 1	2024-06-01	Recruiting	21	Senti Biosciences	NCT06325748
CD5	T-Cell Leukemia, unspecified, T-Cell Lymphoma, unspecified, Mantle Cell Lymphoma, Chronic Lymphocytic Leukemia	Cord Blood	Phase 1/2	2023-11-30	Recruiting	48	M.D. Anderson Cancer Center	NCT05110742
CD7	T-Cell Leukemia, unspecified, T-Cell Lymphoma, unspecified,	NK-92	Phase 1/2	2016-03-01	Unknown	10	PersonGen BioTherapeutics (Suzhou) Co., Ltd	NCT02742727
CD70	B-Cell Lymphoma, unspecified, Myelodysplastic Syndromes, Acute Myeloid Leukemia	Cord Blood	Phase 1/2	2022-11-01	Recruiting	94	M.D. Anderson Cancer Center	NCT05092451
CLL1	Acute Myeloid Leukemia	iPSC	Phase 1	2023-09-07	Recruiting	24	Zhejiang University	NCT06027853
CLL1	Acute Myeloid Leukemia	Peripheral Blood	Phase 1	2024-03-12	Recruiting	26	Shanghai General Hospital, Shanghai Jiao Tong University School of Medicine	NCT06307054
NKG2DL	Acute Myeloid Leukemia, Myelodysplastic Syndromes	Peripheral Blood	Phase 1	2020-09-21	Active, not recruiting	61	Nkarta Inc	NCT04623944
NKG2DL	Acute Myeloid Leukemia	Cord Blood	Undisclosed	2021-10-13	W/T/S	9	Hangzhou Cheetah Cell Therapeutics Co., Ltd	NCT05247957
NKG2DL	Acute Myeloid Leukemia	Undisclosed	Undisclosed	2023-03-01	Recruiting	30	Zhejiang University	NCT05734898
NKG2DL	Acute Myeloid Leukemia	Peripheral Blood	Phase 1	2023-09-21	Recruiting	18	Tongji Hospital, Tongji Medical College, Huazhong University of Science and Technology	ChiCTR2300076016
NKG2DL	Multiple Myeloma	Undisclosed	Early Phase 1	2024-04-22	Not yet recruiting	9	Changzhou No.2 People’s Hospital	NCT06379451
TIM3/CD33	Acute Myeloid Leukemia	Cord Blood	Phase 1	2021-02-04	Recruiting	30	Xiang’an Hospital Affiliated to Xiamen University	ChiCTR2100043081
Undisclosed	Acute Lymphoblastic Leukemia, B-Cell Lymphoma, unspecified	Undisclosed	Phase 1/2	2021-01-29	Unknown	12	Nanfang Hospital, Southern Medical University	NCT04747093
Undisclosed	B-Cell Lymphoma, unspecified, Acute Lymphoblastic Leukemia	Undisclosed	Early Phase 1	2024-02-10	Active, not recruiting	10	The Second Hospital of Shandong University	NCT06596057

**Table 3:** Clinical trials of CAR NK cell agents for autoimmune and infectious diseases

Target	Disease	Cell source	Phase	First posted	Current status	Enrollment	Sponsors	Identifier
NKG2DL/Spike	COVID-19	Cord Blood	Phase 1/2	21/02/2020	Unknown	90	Chongqing Public Health Medical Center	NCT04324996
CD19	Systemic Lupus Erythematosus (SLE)	Undisclosed	Early Phase 1	24/08/2023	Recruiting	12	Changhai Hospital	NCT06010472
CD19	Systemic Lupus Erythematosus (SLE)	Undisclosed	Phase 1	04/01/2024	Recruiting	55	RenJi Hospital	NCT06208280
CD19	Autoimmune diseases, unspecified	Undisclosed	Early Phase 1	13–03–2024	Recruiting	15	Affiliated Hospital of Jiangsu University	NCT06318533
CD19	Primary Immune Thrombocytopenia	Undisclosed	Early Phase 1	30/03/2024	Not yet recruiting	9	Changzhou No.2 People’s Hospital	NCT06337474
CD19	Lupus nephresis	Cord Blood	Phase 1	17/4/2024	Not yet recruiting	20	Takeda	NCT06377228
CD19	Systemic Lupus Erythematosus	Undisclosed	Phase 1	17/5/2024	Recruiting	10	Second Affiliated Hospital, School of Medicine, Zhejiang University	NCT06421701
CD19	Lupus nephresis	Peripheral Blood	Phase 1	13/06/2024	Recruiting	21	Nkarta Inc	NCT06557265
CD19	Relapsed/​Refractory Immune Nephropathy	Undisclosed	Early Phase 1	21/06/2024	Recruiting	36	Changhai Hospital	NCT06469190
CD19	Autoimmune diseases, unspecified	Undisclosed	Phase 1	27/06/2024	Recruiting	72	Changhai Hospital	NCT06464679
CD19	Systemic Lupus Erythematosus	Peripheral Blood	Phase 1	01/07/2024	Recruiting	6	Nkarta Inc	NCT06518668
CD19	Systemic Lupus Erythematosus (SLE), Lupus nephresis	iPSC	Phase 1	01/08/2024	Recruiting	30	Century Therapeutics, Inc	NCT06255028
CD19	Antisynthetase Antibody Syndrome, Rheumatoid Arthritis	Undisclosed	Early Phase 1	20/10/2024	Not yet recruiting	24	The First Affiliated Hospital with Nanjing Medical University	NCT06613490
CD19	Autoimmune diseases, unspecified	Undisclosed	Undisclosed	01/11/2024	Not yet recruiting	15	Second Affiliated Hospital, School of Medicine, Zhejiang University	NCT06614270

**Fig. 1:**
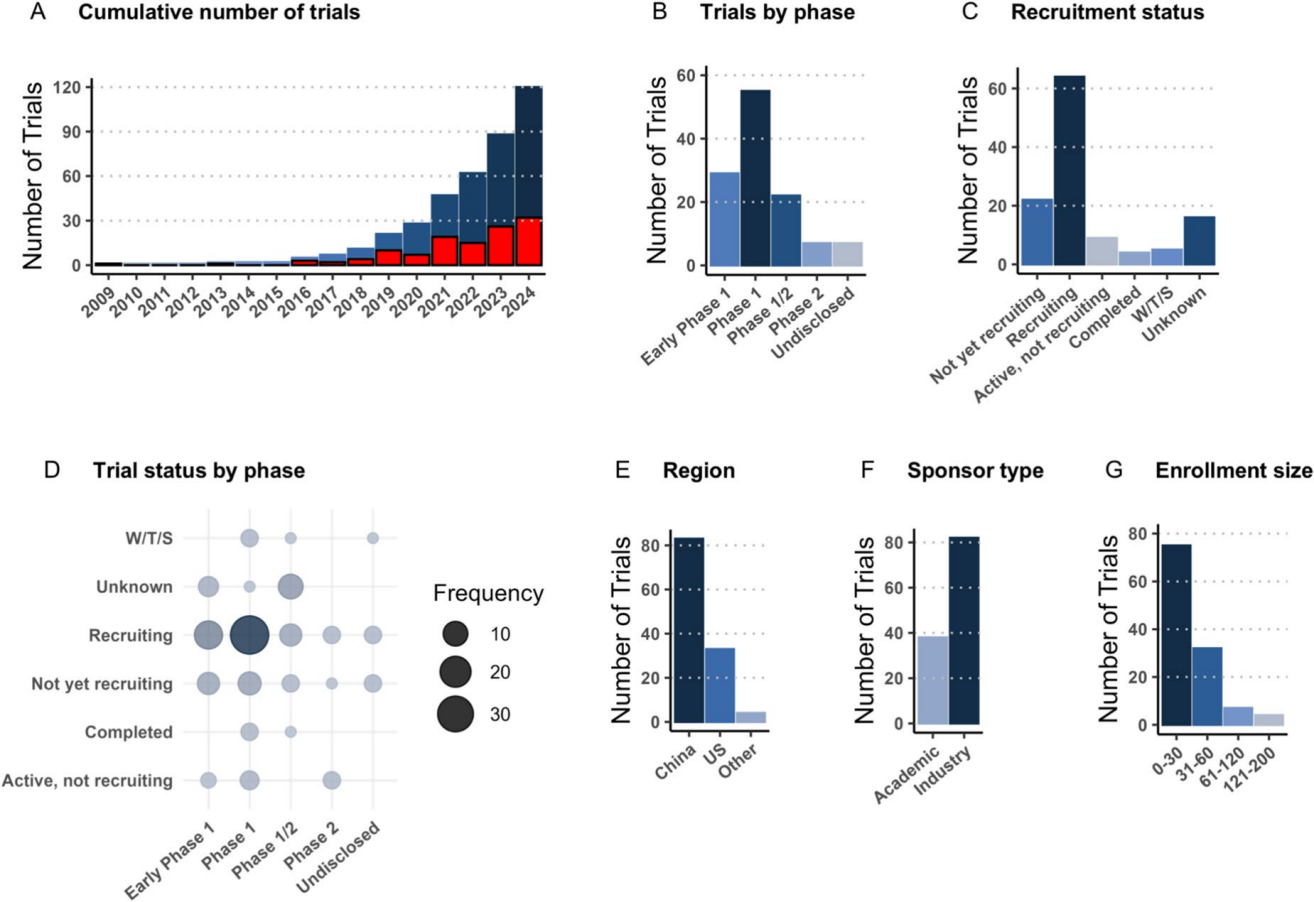
Overview of CAR NK clinical trial characteristics. **A** Total number of registered trials across the US, EU, China, and Japan, including withdrawn, terminated, or suspended trials, but excluding trials that never enrolled patients; red bars represent annual registrations. **B** Classification of trials by phase, with some entries unspecified on clinicaltrials.gov. **C** Recruitment statuses. **D** Trial status by phase, with increasing bubble size indicating higher frequency. **E** Geographical origin of trial sponsors. **F** Type of sponsorship, delineating between academic and industry-involved trials. **G** Range of enrollment sizes showcased, not differentiating between actual and anticipated participant numbers. *US* United States, *W/T/S* withdrawn/terminated/suspended.

The details and characteristics of registered clinical trials are summarized in Fig. 1. The first registered trial investigating CAR NK cell therapy was conducted by St. Jude Children’s Research Hospital in 2009 (NCT00995137). Since then, at least 119 additional trials have been initiated worldwide, with 26 trials in 2023 alone, and 32 so far in 2024 (Fig. 1A). The majority of the trials are currently recruiting or yet to recruit, with a large number of the trials being in phase 1 (Fig. 1B–D). A handful of these studies have advanced to phase 2 (NCT05020015, NCT06161545, NCT06061809, among others), indicating a progression in the clinical development of CAR NK therapies, though results are still forthcoming. The geographical distribution of the trials reveals a concentration in China and the United States, with about two-thirds conducted in China and one-third conducted in the U.S. (Fig. 1E). Around two-thirds of the trials are backed by corporate sponsors, while academic institutions sponsor the remaining one-third (Fig. 1F). This mix underscores the broad appeal of CAR NK cell therapies, attracting both commercial investment and academic research efforts. While early-phase studies typically involve smaller cohorts, usually recruiting fewer than 30 patients, there are notable exceptions, as a few studies are currently anticipating the enrollment of more than a hundred patients (NCT05678205, NCT05950334, NCT05020678, among others) (Fig. 1G).

### Targets and indications

**Fig. 2:**
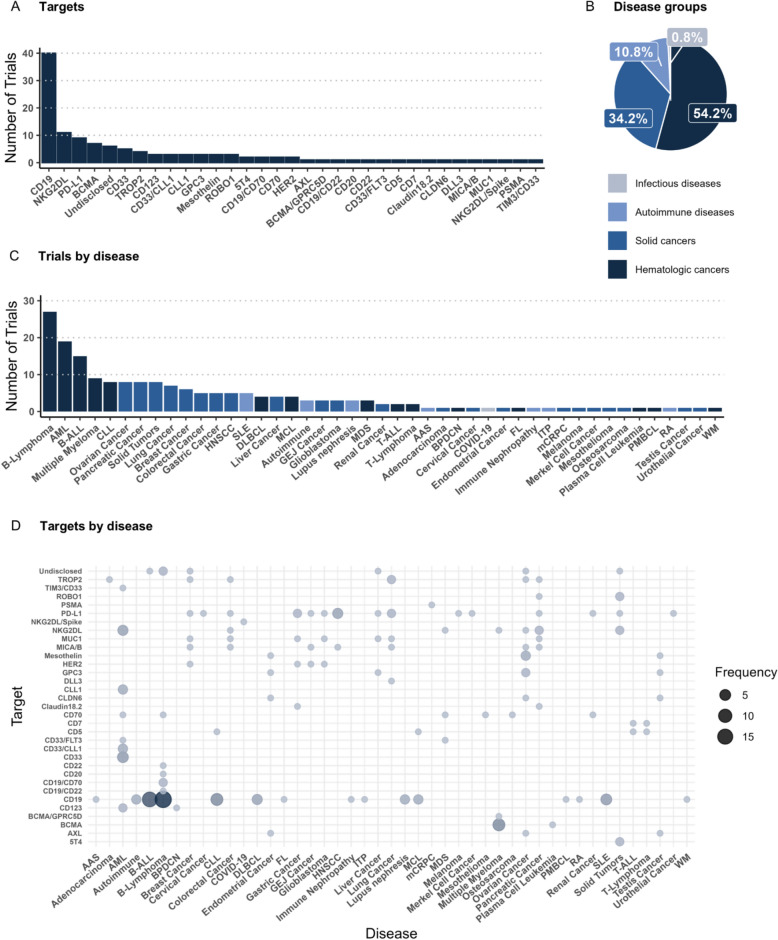
Overview of targets and diseases investigated in clinical trials. **A** Distribution of trials by molecular targets with each trial registering at least one target. Double targets are indicated and separated with “/”. **B** Overall disease groups that are addressed throughout the clinical trials. **C** Diseases targeted by CAR NK cell therapies^1, and colored by disease group. Note that multiple diseases can be registered for one trial. Trials terminated before first patient-enrollment are not included in the figures. **D** Targets distributed across the different diseases, with increasing bubble size indicating higher frequency. *B-Lymphoma* B cell Lymphoma (unspecified), *AML* Acute myeloid leukemia, *B-ALL* B cell Acute Lymphoblastic Leukemia, *CLL* Chronic Lymphocytic Leukemia, *DLBCL* Diffuse Large B cell Lymphoma, *MCL* Mantle Cell Lymphoma, *HNSCC * Head and Neck Squamous Cell Carcinoma, *SLE* Systemic Lupus Erythematosus, *FL* Follicular Lymphoma, *GEJ Cancer* Gastroesophageal Junction Adenocarcinoma, *MDS* Myelodysplastic Syndromes, *Autoimmune* Autoimmune diseases, unspecified, *T-ALL* T cell Acute Lymphoblastic Leukemia, *T-Lymphoma* T cell Lymphoma, unspecified, *BPDCN* Blastic Plasmacytoid Dendritic Cell Neoplasm, *ITP* Primary Immune Thrombocytopenia, *mCRPC* Metastatic Castration-Resistant Prostate Cancer, *MZL* Marginal Zone Lymphoma, *PMBCL* Primary Mediastinal Large B cell Lymphoma, *WM* Waldenströms Macroglobulinemia.

Among the 120 trials, we identified 25 distinct CAR targets and seven unique target pairs (Fig. 2A). These trials encompassed a broad spectrum of 36 different diseases, including hematologic cancers (54.2%), solid cancers (34.2%), autoimmune disorders (10.8%), and infectious diseases (0.8%) (Fig. 2B–D).

### Hematologic cancers

Various types of hematologic cancers, many of which overlap with those pursued for CAR T therapies, are currently under clinical investigation for CAR NK cell therapy. Prominent among these are B cell lymphomas, including diffuse large B cell lymphoma (DLBCL), follicular lymphoma (FL), mantle cell lymphoma (MCL), and marginal zone lymphoma (MZL). Leukemias such as chronic lymphocytic leukemia (CLL) and acute lymphoblastic leukemia (ALL) are also being targeted, with at least 40 trials focusing on CD19 as the primary target. Additionally, CD20 and CD22 are being targeted due to their high expression on B cells [17, 18]. Acute Myeloid Leukemia (AML) is another extensively researched hematologic cancer, accounting for 18% of the total leukemia cases worldwide [19]. In clinical studies, CAR NK cell therapies are directed at various targets, including CD33, CLL1, CD123, and CD70. Additionally, bispecific approaches are gaining traction, with combinations such as CD33/CLL1, CD33/FLT3, and CD33/TIM3 being investigated for enhanced therapeutic efficacy. T cell lymphomas present unique challenges for CAR T cell therapy due to the shared expression of target antigens between malignant T cells and CAR T cells, increasing the risk of fratricide during both production and in vivo application [20]. Current efforts are focused on targets like CD5 and CD7 to address these complex malignancies, although these targets are also expressed on NK cells, thus necessitating their functional knock-out (KO), often achieved through techniques like CRISPR [21, 22]. Multiple myeloma (MM) is another major hematologic malignancy, with over 160,000 new cases diagnosed globally each year [23]. In clinical trials using CAR NK cells, the primary target of interest is BCMA, which is predominantly expressed on plasma cells and a subset of mature B cells, with minimal expression in hematopoietic stem cells or non-hematopoietic tissue [24]. A bispecific approach, targeting both BCMA and GPRC5D, is also currently being investigated using NK cells. Two CAR T cell therapies are currently approved for treatment of MM by targeting BCMA, thus validating this target [2]. Finally, several other hematologic cancer types are also the focus of clinical CAR NK cell therapy development. These include blastic plasmacytoid dendritic cell neoplasm (BPDCN), targeted through CD123; Waldenströms macroglobulinemia (WM), addressed through the NKG2D ligand (NKG2DL); and myelodysplastic syndromes (MDS), which are targeted using CD70.

### Solid cancers

Solid cancers  present distinct challenges for adoptive cell therapies, largely due to the harsh, immunosuppressive tumor microenvironment (TME) [25, 26]. At least 40 clinical studies with CAR NK cells targeting solid cancers have been registered to date. Pancreatic cancer remains one of the most challenging cancers to treat and is the focus of multiple ongoing clinical trials exploring CAR NK cell therapies. Pancreatic ductal adenocarcinoma (PDAC), in particular, is one of the deadliest forms, with a 5-year survival rate of less than 10% [27]. Key targets under clinical investigation for pancreatic cancers include PD-L1, an immune checkpoint ligand and ROBO1, a transmembrane receptor from the Ig superfamily. Other targets include TROP2 and MUC1, both glycoproteins frequently overexpressed in solid tumors; Claudin18.2, a tight-junction molecule; and the stress-induced ligands of the NKG2DL group, such as MICA/B and ULBP1-6, which are natural ligands of NK cells, and commonly overexpressed on various cancers [28]. Prostate cancer, and particularly metastatic castration-resistant prostate cancer (mCRPC), is another highly challenging type of solid cancer and the most prevalent type of cancer in males and the second most deadly [29]. Prostate-specific membrane antigen (PSMA) is a membrane-bound enzyme that is commonly overexpressed in prostate cancers, and the only target currently pursued clinically with CAR NK cells for this indication. Other therapeutic approaches, such as targeted radioligand therapies and antibody–drug conjugates, have also been developed to target PSMA, providing strong proof of concept for its potential as a therapeutic target [30]. Multiple clinical trials are also looking to treat glioblastoma by targeting MUC1, PD-L1 or HER2 with CAR NK cells. The current first-line standard of care treatment for this indication leads to a median overall survival (OS) of only about 15 months [31]. Furthermore, elevated HER2 protein levels have been associated with poorer survival outcomes in this indication, highlighting the potential importance of targeting HER2 [32]. In addition, several other solid cancer indications are being explored in clinical trials using CAR NK cells, targeting antigens such as 5T4, CD70, mesothelin, CLDN6, DLL3, AXL, and GPC3.

### Autoimmune diseases

Autoimmune diseases are a diverse group of chronic inflammatory disorders in which the immune system mistakenly targets the body’s own cells, tissues, or organs. Among various immunological mechanisms, B cells frequently play a central role by producing autoantibodies, presenting antigens to T cells, and releasing pro-inflammatory cytokines. Recently, CAR T cells have been used successfully in treating refractory systemic lupus erythematosus (SLE) by targeting pathogenic B cells. In one study, eight patients received CD19-directed CAR T cells, resulting in complete SLE remission and the maintenance of a Disease Activity Index (SLEDAI) of 0 without immunosuppressive drugs at the three-month follow-up [33]. Given these encouraging outcomes, researchers are actively exploring off-the-shelf solutions like CAR NK cells to treat autoimmune diseases. Notably, out of 32 clinical studies registered for CAR NK cells so far during 2024, 12 of them are investigating autoimmune diseases. Five studies are registered to investigate CAR NK cells in SLE, all of them targeting CD19 (NCT06010472, NCT06208280, among others). Other indications under active investigation include primary immune thrombocytopenia (ITP), immune-related nephropathy, and lupus nephritis (NCT06337474, NCT06469190, NCT06377228), all of which involve targeting B cells via CD19.

### Infectious diseases

Moreover, CAR NK cell therapies have also been explored in a clinical context for infectious diseases. In 2020, a clinical trial was registered to investigate an NKG2DL/Spike-directed bispecific CAR NK cell therapy for COVID-19, seemingly by incorporating ACE2 and NKG2D extracellular domains into the CAR (NCT04324996). However, the current status of the trial remains unknown, and no public results have been reported thus far.

## Genetic modifications of CAR NK cells

**Fig. 3:**
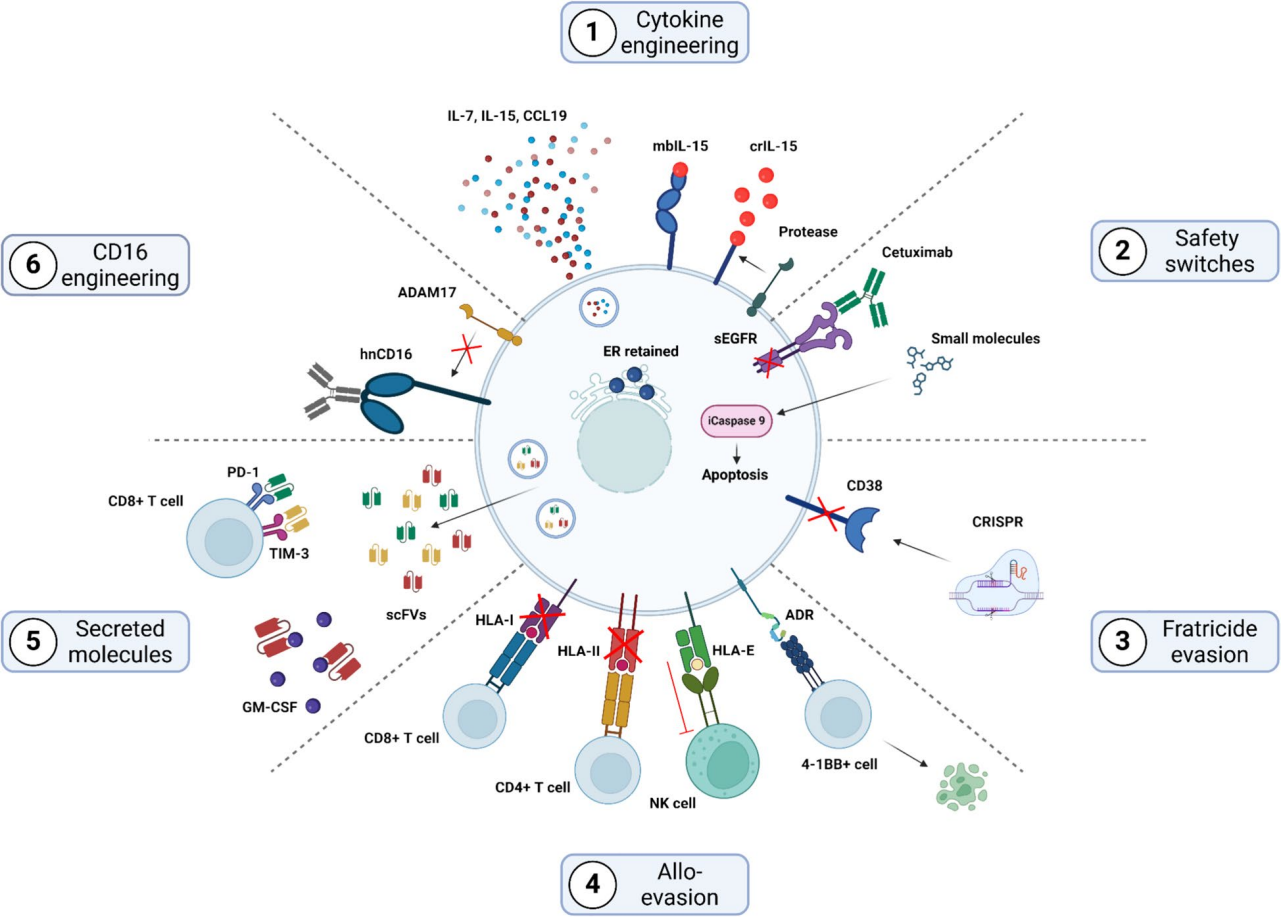
Schematic overview of the genetic modifications in CAR NK cells. **1** Cytokine modifications for enhanced function. **2** The integration of a safety switch mechanism for controlled apoptosis and targeted cell elimination via ADCC for improved safety. **3** Gene KO to facilitate fratricide evasion. **4** Allo-evasion techniques including HLA modifications. **5** Secretion of regulatory molecules for immune modulation. **6** Engineered CD16 for improved targeting and cytotoxicity. *PD-1* Programmed cell death protein 1, *TIM-3* T-cell immunoglobulin and mucin-domain containing-3, *GM-CSF* Granulocyte–macrophage colony-stimulating factor, *HLA-I* Human leukocyte antigen-I, *HLA-II* Human leukocyte antigen-II, *HLA-E* Human leukocyte antigen-E, *ADR* alloimmune defense receptor, *mbIL-15* membrane bound IL-15, *ADAM17* A disintegrin and metalloprotease 17, *hnCD16* high-affinity, non-cleavable CD16.

While CAR NK cells may induce effective killing of target cells, many challenges persist in achieving long lasting, durable responses. To overcome these challenges, a variety of different genetic modifications have been introduced to enhance cytotoxicity, survival and persistence of CAR NK cells in vivo. A subset of these modifications is currently being tested in clinical trials, with Fig. 3 providing a schematic overview of the modifications.

### Cytokine engineering

Multiple trials are testing CAR NK cells that have been genetically engineered to either secrete recombinant cytokines or express membrane-bound versions of different cytokines. The most widely studied cytokine for enhancement of CAR NK cell therapies is IL-15. The cytokine activates NK cells through a receptor complex composed of IL-2/IL-15 receptor beta (β) chain and the common gamma chain (γc) and promotes survival, proliferation, and cytotoxicity of the NK cells [34, 35]. In some studies (e.g. NCT05336409 and NCT04324996), CAR NK cells were engineered to secrete IL-15 or agonists for the IL-15 receptor systemically, which has been shown to augment the anti-tumor effects of CAR NK cells in preclinical models and also modulate the TME [36, 37]. However systemic secretion of IL-15 has also been associated with toxicities in preclinical studies [38]. Therefore, membrane-bound IL-15 (mbIL-15), comprising an IL-15/IL-15 receptor alpha (α) fusion protein (IL-15RF), has emerged as a potentially safer alternative (e.g. NCT05020678 and NCT05950334). This approach confines NK cell activation to direct contact, effectively reducing systemic exposure and the unintentional activation of bystander immune cells [39]. Additionally, two other studies employ calibrated release of IL-15 (crIL-15) in which membrane bound IL-15 is cleaved from the surface of CAR NK cells by proteases, ensuring localized release only (NCT06325748 and NCT06652243) [40, 41].

IL-2 is also considered to be a survival factor for NK cells; however, the systemic administration of this cytokine has been associated with severe complications, partly due to the uncontrolled stimulation of effector cells and partly due to the potential for stimulating growth of regulatory cells, such as regulatory T cells [42]. Retaining IL-2 in the endoplasmic reticulum (ER) has been investigated as an approach to mediate autocrine signaling with this cytokine, while avoiding the potential side-effects attributed to exogenous administration [43]. This modification has been used in various studies, primarily involving CAR-modified NK-92 cell lines (NCT03228667, NCT05618925, NCT04847466, NCT04052061, among others). In another clinical study (NCT05410717), CAR NK cells were engineered to express and secrete IL-7 and CCL19. IL-7 has been shown to enhance survival of human CD56^bright^ NK cells, potentially improving the persistence of the CAR NK in vivo [44]. CCL19 is a C–C chemokine which binds and activates CCR7, a receptor that is typically highly expressed on NK cells and other effector immune cells. It functions as a chemotactic receptor, usually involved in homing of these cells to secondary lymphoid tissues. However, for the aforementioned clinical study, it is likely used for targeted recruitment of effector cells into the TME, thereby enhancing an endogenous response against the tumor as well. This approach has also been tested for use in CAR T cells [45].

### Safety switches

Due to random integration of CAR-bearing vectors, addressing the risks of insertional mutagenesis and potential oncogenic transformation is crucial to avoid uncontrolled activation and expansion of CAR NK cells in vivo. Conditional safety switches integrated into the cells offer a solution by enabling the selective elimination of CAR NK in patients reversing any adverse effects, as is also explored in T cells [46]. An example of this strategy is the use of a truncated epidermal growth factor receptor (tEGFR) variant in the clinical trial NCT05336409. This variant, containing the cetuximab binding epitope, serves as a safety switch and has been shown to mediate efficient ablation in vivo of adoptively transferred cells [47]. Cetuximab, a clinically available mAb, can mediate antibody-dependent cellular cytotoxicity (ADCC) against NK cells engineered with this EGFR variant. Thus, it allows for the targeted destruction of CAR NK cells. Another innovative strategy is seen in trials NCT03056339 and NCT05020015. These studies introduce an inducible caspase-9-based suicide gene (iCasp9) into the CAR design of CAR NK cells. The iCasp9 component acts as a safety switch, enabling pharmacologically mediated apoptosis of the CAR NK cells upon detection of adverse responses. This mechanism provides an added layer of safety by allowing for the rapid and targeted elimination of the CAR NK cells if necessary, as shown in CAR T cells [48, 49].

### Fratricide evasion

KO strategies play a promising role in enhancing the efficacy of CAR NK cell therapies in clinical trials. As an example, one study is exploring the impact of CD38 gene KO (NCT05950334) [50]. This modification mitigates the risk of fratricide when combined with CD38-targeted monoclonal antibodies (mAbs) in vivo, as CD38 is also expressed on NK cells. Furthermore, this modification has shown promising results in preclinical studies, significantly bolstering the resilience of CAR NK cells, especially under oxidative stress conditions commonly found in the TME. The attenuation of CD38 expression is anticipated not only to improve the survival and persistence of CAR NK cells but also to amplify their effector functions against cancer cells [51, 52].

### Allo-evasion strategies

As most of the currently ongoing clinical trials are pursuing an off-the-shelf allogeneic approach for the CAR NK cells, a critical challenge is the host immune system’s tendency to recognize and eliminate grafted cells. This recognition is largely due to the presence of non-self molecules presented by HLA-I and HLA-II on the allogeneic CAR NK cells. As a result, these cells often face limited persistence in vivo, leading to minimal therapeutic response or faster relapse [53]. Several innovative allo-evasion strategies are currently under clinical investigation to address this challenge. One such approach being tested in clinical studies (NCT05336409 and NCT06255028) involves the strategic modification of the composition of HLA molecules on the CAR NK cells by knocking out HLA-I and HLA-II, which aims to reduce their recognition by CD8 + and CD4 + T cells, respectively. Additionally, a knock-in of HLA-E is performed to evade detection by host NK cells, which typically attack cells lacking HLA class I [54]. Another promising strategy is explored in a study (NCT05950334), where researchers are employing a novel alloimmune defense receptor (ADR) technology. This involves a synthetic engineered receptor that targets 4-1BB expressed on alloreactive immune cells such as the host T cells and NK cells, to eliminate these cells. In preclinical studies, the engagement of ADR-armed CAR NK cells with alloreactive immune cells has been shown to mitigate rejection, promote cellular proliferation, and enhance anti-tumor activity. This indicates that ADR-armed CAR NK cells could be effective without the need for intensive conditioning chemotherapy [55].

### Other secreted molecules

Another innovative strategy focuses on countering the immunosuppressive TME to enhance the antitumor response by secretion of inhibitory molecules by the CAR NK cells. This approach is exemplified in the study NCT05410717, where CAR NK cells are genetically modified to secrete single-chain variable fragments (scFvs). These scFvs specifically target the inhibitory receptors PD-1, CTLA-4, and LAG-3, which are found on NK cells and various other effector immune cells, including CD8 + T cells [56, 57]. By binding to and blocking these inhibitory receptors, the secreted scFvs effectively disrupt the immunosuppressive signals, thereby enhancing the cytotoxic activity of both CAR NK cells and other immune effector cells within the TME [58]. This approach has been validated in solid tumor models with HER2-targeted CAR NK cells [59]. In another study (NCT04324996), CAR NK cells were engineered to secrete scFvs targeting granulocyte–macrophage colony-stimulating factor (GM-CSF). This approach aims to neutralize GM-CSF, which has been shown to be implicated in the adverse events of CRS and immune effector cell-associated neurotoxicity syndrome (ICANS), thus enhancing the safety profile of CAR NK cell therapy [60].

### CD16 engineering

Another approach to enhance the CAR NK cell cytotoxicity, currently being tested in multiple clinical trials (NCT04390399, NCT05950334, NCT05182073 among others), is the engineering of high-affinity, non-cleavable CD16 (hnCD16) Fc receptors. This modification aims to enhance the natural ability of the CAR NK cells to mediate ADCC against tumor cells. CD16, which is also known as FcγRIII, is a receptor expressed on the surface of NK cells, macrophages, and some subsets of T cells. The engagement of CD16 with the Fc region of antibodies leads to the activation of effector cells and the subsequent killing of antibody-coated target cells through ADCC [61]. The novel hnCD16 Fc receptor has been engineered with two key modifications: increased affinity for the Fc region of antibodies and resistance to cleavage and down-regulation upon activation. The F158V variant of hnCD16 exhibits a higher affinity for the Fc region of IgG antibodies [62]. This enhanced affinity ensures more stable interactions with therapeutic mAbs, augmenting the efficiency of the ADCC process. Moreover, under normal physiological conditions, the CD16 receptor can be cleaved from the NK cell’s surface upon activation, which leads to a reduction in the cell’s ability to mediate ADCC. The hnCD16 receptor is engineered to be non-cleavable, maintaining its expression on the cell surface and sustaining the cytotoxic capability over time. This ensures a sustained immune response against tumor cells, especially in the repetitive or prolonged exposure to target antigens and therapeutic mAbs [61, 62].

## Cell sources

**Fig. 4:**
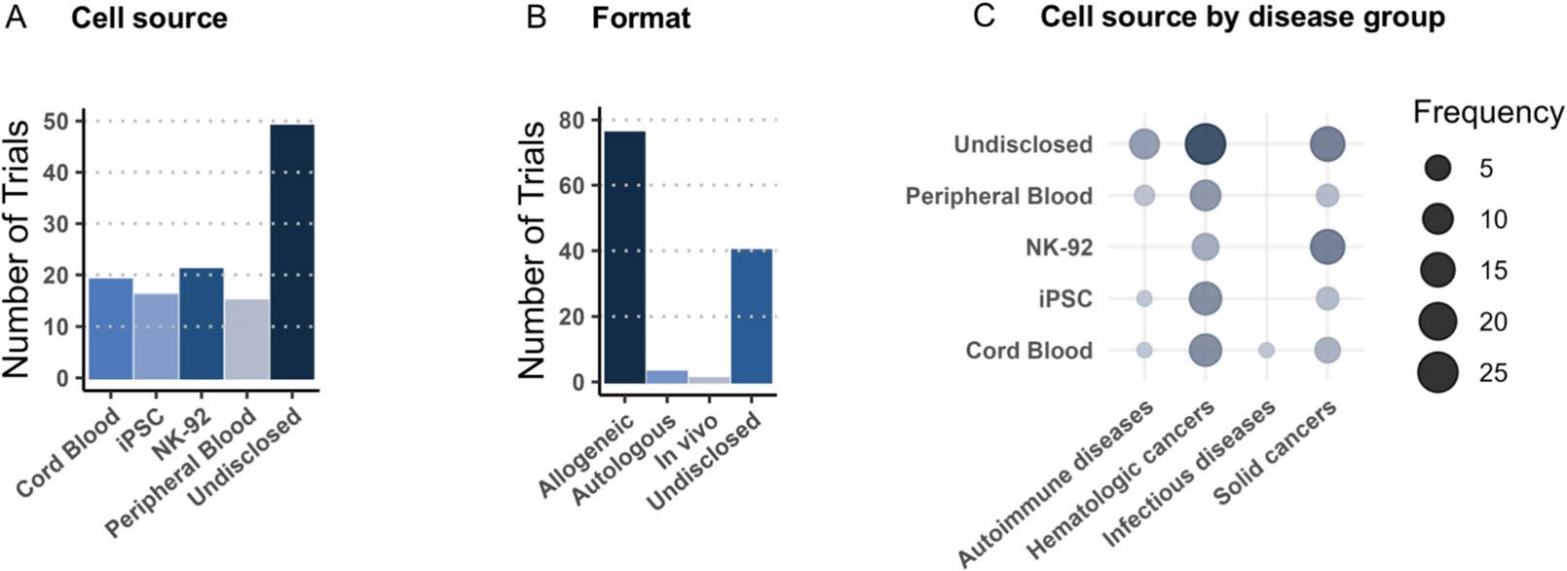
Overview of cell sources and formats investigated in clinical trials. **A** The figure summarizes the sources from which the NK cells are derived. **B** Distribution of clinical trials based on whether the NK cells are administered as allogeneic or autologous products. **C** Cell sources distributed across the different disease groups, with increasing bubble size indicating higher frequency. *iPSCs* inducible pluripotent stem cells.

One of the major advantages of CAR NK cell therapy over CAR T cells is the ability to harness NK cells from a variety of sources. NK cells can be obtained from both autologous and allogeneic sources, each offering distinct benefits and challenges. Here, we describe some of the advantages and drawbacks of the NK cell sources currently being explored in clinical trials. It is worth noting, that the distribution of cell sources employed in currently registered clinical trials is fairly even, indicating that the optimal starting material, if any, is yet to be determined (Fig. 4A).

### Peripheral blood NK cells

Peripheral blood NK cells (PB-NK) can be harvested directly from peripheral blood by leukapheresis and represent an easily accessible and readily available source of NK cells [63]. PB-NK cells are phenotypically mature and express a broad range of cytotoxicity receptors, making them highly cytotoxic against malignant cells [64]. PB-NK cells can be isolated from either the patient (autologous) or healthy donors (allogeneic). Allogeneic PB-NK cells are generally considered more favorable in clinical contexts compared with their autologous counterpart, as they enable “off-the-shelf” therapies and are more resistant to immune silencing induced by self-MHC molecules presented on malignant cells [65, 66]. Moreover, allogeneic NK cells derived from healthy donors may be of superior quality compared with autologous NK cells from patients that may have undergone rounds of lymphodepleting treatments. These benefits are reflected by the vast majority of registered clinical trials using allogeneic cell sources for CAR NK cell therapy (Fig. 4B). To date, at least 15 clinical trials have employed PB-derived CAR NK cells, with seven using allogeneic sources, three using autologous cells, and five remaining undisclosed.

However, challenges persist in producing large quantities of clinical-grade CAR NK cells, as NK cells make up only about 10% of peripheral blood lymphocytes, often necessitating ex vivo expansion [67]. However, lack of standardized protocols used to activate and expand NK cells, as well as inter-donor variability, can lead to heterogeneity of the CAR NK cell products [68]. In addition, genetic modification of PB-NK cells using lentiviral vectors is difficult using established protocols from CAR-T production, likely due to innate antiviral defenses. One approach currently being explored to enhance NK cell transduction efficiency involves altering the tropism of the lentiviral delivery system using alternative lentiviral pseudotypes [69].

### Cord blood NK cells

Cord blood NK cells (CB-NK) represent another common starting material for CAR NK cell products. CB-NK cells make up approximately 30% of all lymphocytes in cord blood and generally exhibit a more naive phenotype compared with PB-NK cells [67, 70]. CB-NK cells also exhibit a higher proliferation capacity and they are less resistant to genetic editing than PB-NK cells, making them suitable for large-scale production of CAR NK cells. However, CB-NK cells express fewer cytotoxicity receptors and are subsequently less cytotoxic than mature PB-NK [70]. In addition, the limited volume that can be obtained from a cord blood sample demands extensive ex vivo expansion of NK cells to achieve clinically relevant doses [71]. Nevertheless, more than 100 doses of CAR NK cells have been manufactured from a single cord blood sample [72]. To date, at least 20 clinical trials have utilized cord blood derived CAR NK cells.

### NK cell lines

NK cell lines are also being explored in clinical trials, due to their ability to proliferate indefinitely, enabling large numbers of CAR NK cells to be generated quickly. The NK cell line, NK-92, has been explored in at least 22 clinical trials and represents the most common starting material for CAR NK cell products in clinical trials. NK-92 cells are derived from a patient with aggressive NK cell lymphoma and display features of naive NK cells but are highly cytotoxic, reflected by a high expression of activating receptors and low levels of inhibitory receptors [73, 74]. In addition, the unlimited proliferation capacity of NK-92 cells combined with the ability to introduce multiple genetic edits, enable generation of stable NK-92 cell clones with enhanced therapeutic potential [75]. However, infusion of immortal NK cell lines raises several safety concerns related to the risk of secondary tumor engraftment. As a safety measure, NK-92 cells have to be irradiated before use, which negatively impacts their long-term persistence and potency [76]. Interestingly, NK-92 cells are currently being used as a starting material in the treatment of primarily solid cancers, such as HNSCC, glioblastoma, and lung cancer (Fig. 4C).

### Stem cell-derived NK cells

Pluripotent stem cells represent another promising source of “off-the-shelf” CAR NK cells, and have so far been used in at least 17 clinical trials. Stem cell-derived NK cells are commonly sourced from induced pluripotent stem cells (iPSCs) or human embryonic stem cells (hESCs) that have the ability to grow indefinitely in their undifferentiated state. These progenitor cells can then be genetically modified and subsequently differentiated into mature NK cells [77]. Manufacturing of stem cell-derived NK cells typically involves the use of cytokine cocktails or co-culture with stromal cells, resulting in NK cells with mature expression profiles and robust cytotoxicity, similar to that of PB-NK cells [78, 79]. The ability to consistently develop NK cells from iPSCs and hESCs enables standardized manufacturing strategies with minimal heterogeneity of CAR NK cell products [80]. Similar to the use of NK cell lines, this approach also enables the introduction of multiple genetic modifications to augment the biological activity of CAR NK cells [81]. However, manufacture of stem cell-derived CAR NK cells comes with its challenges. Firstly, differentiation of pluripotent stem cells requires complex systems that limit the ability to cost-effectively scale the production of good manufacturing practices (GMP)-grade CAR NK cells [82, 83]. Secondly, production of large quantities of CAR NK cells is time-consuming, typically taking several weeks, and cultured cells may accumulate oncogenic mutations during the expansion process, posing safety risks [84–86].

### In vivo generated CAR NK cells

A new approach to CAR NK cell therapy involves direct in vivo injection of the gene delivery particles, eliminating the need for ex vivo manipulation of NK cell products entirely. This approach is being used in a trial (NCT06539338) in which lentiviral vectors specifically engineered to target CD7 positive cells, such as T cells and NK cells, are administered to patients diagnosed with NHL. The lentiviral vectors carry a transgene encoding a CAR directed towards CD20, a well known B cell specific antigen [87]. While this approach could potentially provide a low-cost off-the-shelf product, the direct injection of lentiviral particles introduces specific safety concerns, due to the potential risk of insertional mutagenesis and oncogenic transformation, which has been observed with other lentiviral based gene therapies [88]. Additionally, since manufacturing details and safety data are still forthcoming, the full extent of potential risks associated with in vivo delivery remains unknown.

## Manufacturing strategies

**Fig. 5:**
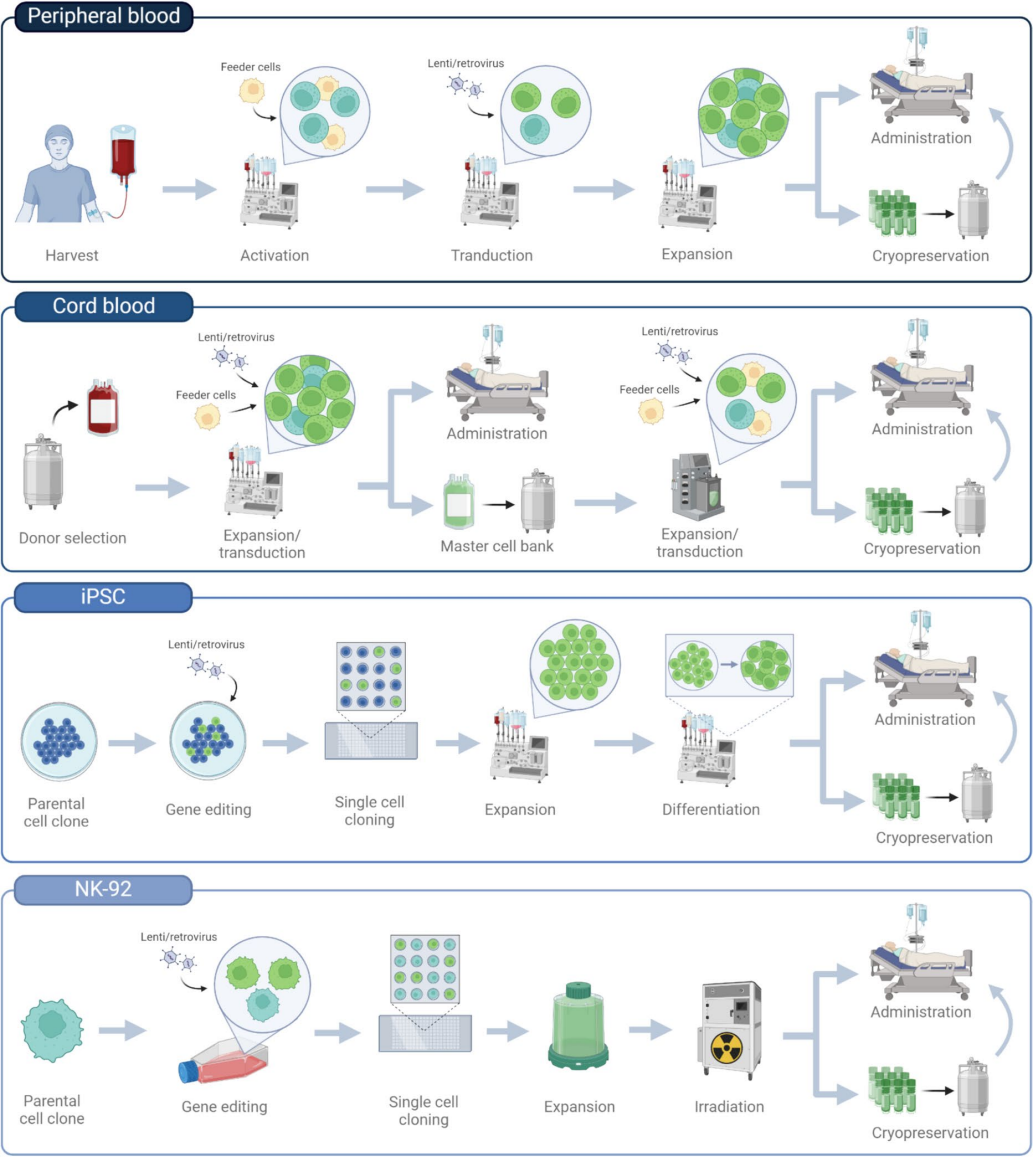
CAR NK cell manufacturing strategies. The protocol used to manufacture CAR NK cells differ depending on the starting material used. The manufacturing steps shown are based on platforms employed by Nkarta, Inc. (peripheral blood NK cells), Artiva Biotherapeutics, Inc./Takeda Pharmaceuticals Ltd. (cord blood NK cells), Fate Therapeutics, Inc. (iPSC NK cells), and academic platforms (NK-92 cells). *iPSC* induced pluripotent stem cell.

Manufacturing of sufficient quality and quantity of CAR NK cells for clinical use is not trivial, and requires robust GMP-compliant protocols. Strategies used to manufacture CAR NK cell therapies differ depending on the starting material used, but also on the platforms and proprietary technologies employed (Fig. 5). In this section, we go through some of the manufacturing strategies employed in the ongoing clinical trials using CAR NK cell therapies.

### PB-NK

PB-derived CAR NK cells are being actively investigated in clinical trials. Nkarta, Inc. has developed a platform for the manufacturing of allogeneic, off-the-shelf NK cell-based therapies (e.g. utilized in NCT05020678). Here, NK cells are collected and isolated from healthy donors by leukapheresis and proprietary feeder cells are used to stimulate NK cell activation and expansion. For genetic engineering of NK cells, Nkarta uses a γ-retrovirus delivery system to introduce the CAR gene, as well as a gene encoding mbIL-15. The modified CAR NK cells are then continuously expanded, followed by harvest and cryostorage of multiple doses [89].

### CB-NK

Artiva Biotherapeutics, Inc., has developed a proprietary manufacturing platform for generating allogeneic CAR NK cells from healthy donor umbilical CB (e.g. utilized in NCT05678205). They start by preselecting donor CB samples expressing the high affinity variant of CD16 (158 V/V) and the killer immunoglobulin-like receptor B (KIR B) haplotype from a registered public CB bank. The selected donor units are CD3 depleted and cultured in plastic bags with modified feeder cells that support NK cell expansion. The expanded NK cells are then genetically engineered to express a HER2-directed CAR and IL-15 by lentiviral transduction, followed by cryopreservation and storage to generate a master cell bank. Each unit from the master cell bank can then be selected for large-scale CAR NK cell expansion and activation using bioreactors, followed by cryostorage for off-the-shelf use.

Another company, Takeda Pharmaceuticals, similarly uses CB-derived CAR NK cells (e.g. utilized in NCT05020015), but differs in their donor selection and manufacturing platform. Here, CB units are pre-selected with KIR ligand mismatch between donor and recipient for CAR NK cell production. KIR ligand mismatch may enhance the intrinsic anti-tumor activity of NK cells through “missing-self” recognition [66, 90]. The CB units are then thawed and CD3-, CD19- and CD14-depleted, followed by co-culture with modified K562 feeder cells expressing membrane-bound IL-21 (mbIL-21) and 4-1BB ligand and exogenous IL-2 to support NK cell growth. The NK cells are then genetically engineered by retroviral transduction to express a CD19-directed CAR, IL-15, and iCasp9. The CAR NK cells are finally expanded further and harvested on day 15 for infusion, without the need for cryopreservation.

### iPSC-NK

A different approach is used by Fate Therapeutics, Inc., as they have developed a proprietary platform for the manufacturing of iPSC-derived CAR NK cells (used in NCT05182073). Here, a multiplexed engineered clonal iPSC master cell bank is used to generate CAR NK cell therapies. They start by establishing an iPSC pool from fibroblasts, which is then genetically engineered to express IL-15RF and an hnCD16 at the CD38 locus for simultaneous CD38 KO [91]. The modified iPSCs are subsequently single-cell cloned to generate a homogenous foundation cell line. The foundation cell line is then subjected to a second round of modifications, which differs depending on the product. Following a second round of single-cell cloning, the multiplexed engineered clonal iPSCs are then stored as a master cell line, and subsequently differentiated into hematopoietic progenitor cells and finally NK cells [91].

### NK-92

Manufacture of clinical-grade CAR NK-92 cell therapies typically involves use of parental NK-92 cells from an FDA-licensed master cell bank as the starting material. The parental cell line is transduced with a lentiviral vector encoding the CAR construct, followed by screening of individual cell clones. The vector integration sites are mapped to identify potential oncogenic insertions. One trial, NCT03383978, investigates the use of HER2-targeted CAR NK-92 cells derived from the well-characterized single-cell clone NK-92/5.28.z [92]. The clonal CAR NK-92 cells can then be expanded using GMP-compliant culture conditions in gas-permeable culture bags. Due to the tumorigenic nature of NK-92 cells, additional steps are taken to prevent engraftment in the patient. Exposure to 10 Gy of γ-irradiation is sufficient to prevent proliferation of NK-92 cells, while maintaining cell potency for 24 h [93]. Depending on the treatment dose, cell concentrations can then be adjusted and the final cell products cryopreserved.

## Preconditioning strategies

**Fig. 6:**
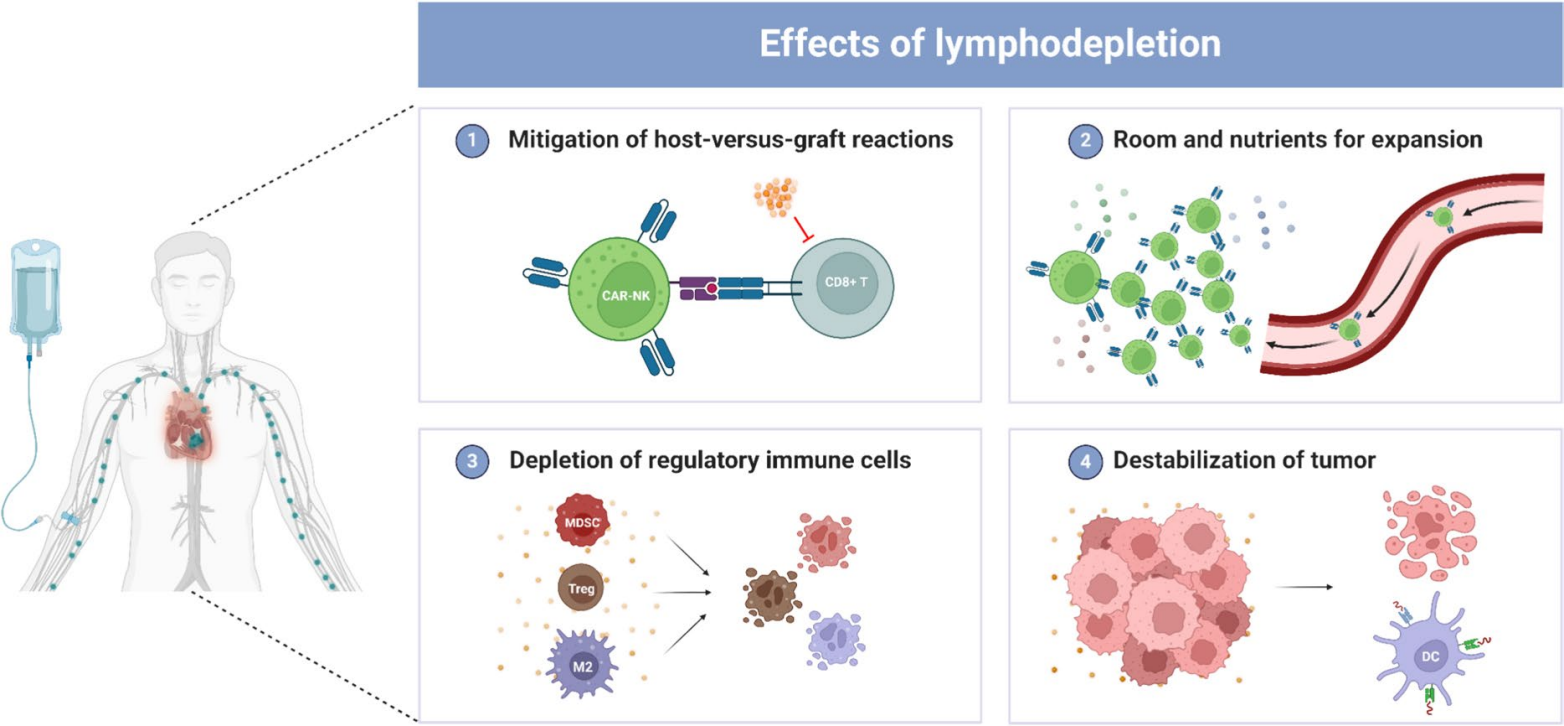
Effects of lymphodepletion. **1** Effector cells, such as CD8 + T cells, are depleted, mitigating host-versus-graft reactions. **2** Lymphodepletion clears space in the circulatory system, providing the essential room and nutrients needed for the expansion of infused CAR NK cells. **3** Regulatory immune cells, including myeloid-derived suppressor cells, regulatory T cells, and M2 macrophages, are depleted from the circulatory system and TMEs, thereby enhancing CAR NK cell activity. **4** The lymphodepletion destabilizes the TME, rendering it more susceptible to the CAR NK cells’ anti-tumor activity. *DC* dendritic cell, *MDSC* myeloid-derived suppressor cells, *M2* M2 macrophage.

Lymphodepletion, a crucial preparatory step in CAR-based therapy, serves to significantly enhance the effectiveness of these treatments [94]. By administering chemotherapy or other lymphodepleting agents before infusing the CAR NK cells, this process systematically reduces the patient’s existing lymphocyte count (Fig. 6).

Importantly, lymphodepletion helps in creating an environment where the CAR NK cells can thrive. By reducing the number of lymphocytes, it ensures that the CAR NK cells have less competition for resources, such as key growth factors, cytokines and nutrients, which allow the infused CAR NK cells to expand more freely [95]. Lymphodepletion also targets regulatory components of the immune system, such as regulatory T cells, M2 macrophages and myeloid-derived suppressor cells (MDSCs) which are known to suppress immune responses, including those mediated by CAR NK cells. Reducing the numbers of these cells can lead to a more active immune environment, especially in the TME [96]. Furthermore, lymphodepleting chemotherapy can cause tumor cell death, leading to increased antigen presentation, e.g. by dendritic cells (DCs). The secretion of various activating cytokines by these cells can subsequently amplify the anti-tumor efficacy of CAR NK cells. Additionally, the application of lymphodepletion in the context of allogeneic cell therapies plays a pivotal role by eliminating cytotoxic immune cells responsible for mediating host-versus-graft reactions. This step improves the in vivo persistence and functional longevity of the infused CAR NK cells [97]. Cyclophosphamide and fludarabine (Cy/Flu) are the predominant lymphodepleting agents, employed in combination in at least 48 out of 54 clinical studies where the lymphodepletion strategy is specified. Cyclophosphamide is an alkylating agent that cross-links DNA, while fludarabine, a purine analogue, inhibits DNA synthesis leading to cell death [98, 99]. Cyclophosphamide is typically administered intravenously at doses of 300 to 500 mg/m^2^ per day over a three-day course, with the dosage on the lower end when used alongside fludarabine. Fludarabine’s standard dose is 25–30 mg/m^2^ per day, given intravenously for three to five consecutive days. Other agents used in these trials for lymphodepletion include bendamustine, cytarabine, decitabine, etoposide, and melphalan. Lymphodepletion, though crucial for enhancing cellular therapies like CAR T and CAR NK, carries downsides including increased infection risk due to immune suppression, prolonged immunodeficiency requiring prophylactic treatments, hematologic toxicity like anemia and leukopenia, and non-hematologic side effects such as nausea and hair loss [94]. For this reason, strategies that may mitigate the need for lymphodepleting regimens are currently being explored. Three studies are currently underway, two with CNTY-101 (NCT05336409 and NCT06255028) and one with FT522 (NCT05950334), with CNTY-101 incorporating HLA-I and HLA-II KOs as well as HLA-E knock-in, and FT522 using the ADR technology previously described.

## Dosing regimens and strategies

Dosing strategies play an important role  in the development and optimization of the clinical potential of CAR NK cells. A diverse range of dosing regimens are currently being tested in clinical trials, reflecting the uncertainties regarding the dose needed to maximize efficacy while minimizing potential adverse effects in patients.

### Dosage size

**Fig. 7:**
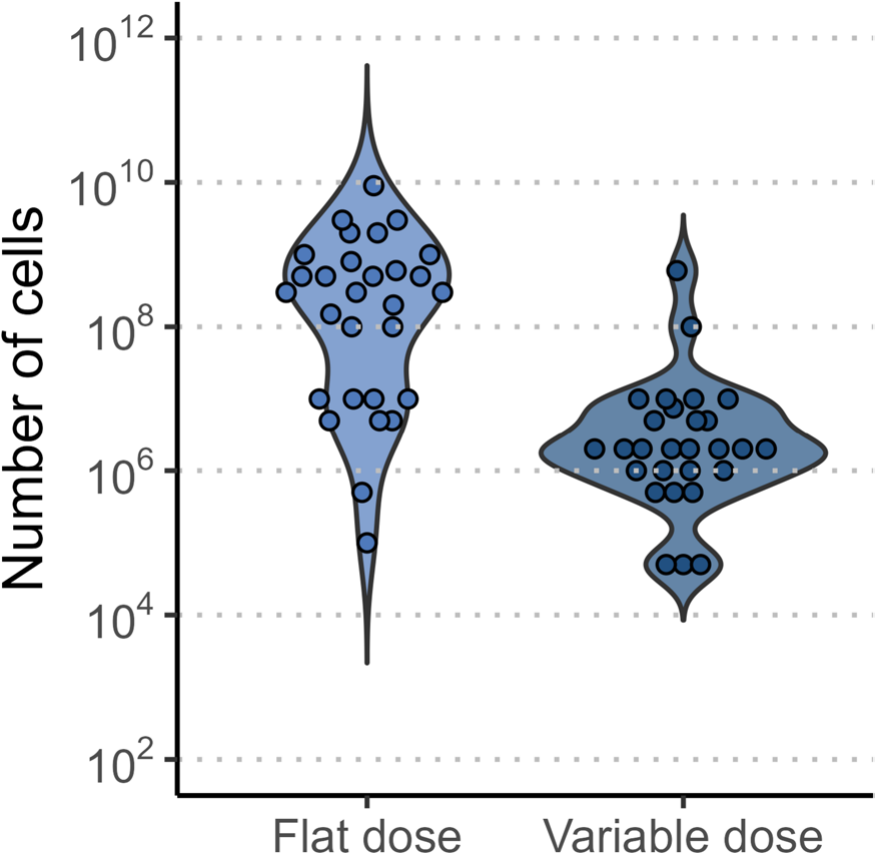
Summary of utilized dosage sizes. The figure presents starting doses as reported on clinicaltrials.gov used during initial dose escalation phases of clinical trials. Dosages are categorized by administration type: flat doses and those varied by recipient weight (cells per kilogram mass).

In early dose escalation studies (phase 1 or phase 1/2), two dosing strategies are currently being tested: a flat dosage size and a variable dosing size based on the weight of the recipient. Figure 7 summarizes the starting doses that were disclosed among the 122 studies at this stage of development, categorized by disease group and cell sources.

For flat dosing, starting doses ranged from 100 thousand to 2 billion cells for hematologic cancers, 1 million to 3 billion cells for solid cancers, and 600 million to 10 billion cells for autoimmune diseases. In the weight-based dosing approach, starting doses ranged from 50 thousand to 800 million cells/kg for hematologic cancers (with 800 million cells/kg appearing to be an outlier); 1 million to 100 million cells/kg for solid cancers; and 5 million to 800 million cells/kg for autoimmune diseases (although only two data points are available for this category).

A notable trend toward higher starting doses is observed in autoimmune disease trials, likely reflecting the hypothesis that a “hard reset” of the immune system is required for these conditions [33]. However, no clear correlation between dosage sizes and cell sources can be drawn from the available data. Only a few studies that have progressed to phase 2 have disclosed dosing information. One example is the study, NCT04847466, which reported a dose of 2 billion cells derived from the NK-92 cell line.

### Dosing schedules

The dosing schedules across the identified clinical trials show a range of strategies, from single to multiple doses. Weekly dosing is common, regardless of cell source, with treatments given once or twice a week, such as for instance on day 0, day 7 and day 14 during a cycle (NCT03656705, NCT05667155, NCT04324996). Some trials administer infusions every 2 weeks, either from the start or after an initial treatment phase, such as after 6 weeks of treatment (NCT05856643, NCT04847466). Furthermore, in some trials, the dose is split in two dosages administered over one day (NCT05247957), or as three smaller weekly dosages (NCT05020678).

## Combination strategies

The advent of CAR NK cell therapies has also fueled the investigation of combination treatments to enhance the functional response of the CAR NK cells. Combinatory treatments function by modulating the activity of the CAR NK cells by increasing cytotoxicity, enhancing survival and persistence or increasing resistance to immune suppression. Current investigations into combination strategies for CAR NK cell treatments can be classified into three main categories: monoclonal antibodies (mAbs), exogenous cytokines, and other small molecules. This section elaborates on the combination strategies currently under investigation in ongoing clinical trials involving CAR NK cells.

### Monoclonal antibodies

Combined administration of mAbs, represents the most common combination strategy for CAR NK cell treatments, and is currently being explored in at least 16 different clinical trials. mAbs can modulate the activity of CAR NK cells through two distinct mechanisms; firstly, mAbs can be used to block immune checkpoint interactions in order to evade immunosuppressive signals produced by malignant cells [100]. Several immune checkpoints have been implicated in immune suppression, with the PD-1/PD-L1 axis being the most common, as PD-L1 is upregulated in many cancers [101]. Hence, mAbs targeting both PD-1 and PD-L1 are actively being investigated in combination with CAR NK cell therapies (NCT03228667, NCT04847466, NCT05395052, NCT03383978, among others). One study (NCT03228667) is currently exploring the therapeutic benefit of five different mAbs targeting PD-1 or PD-L1 in combination with PD-L1-directed CAR NK cells in cancer patients who have relapsed from treatment with immune checkpoint inhibitors. Another study (NCT05395052, now terminated) also investigated CAR NK cells in combination with several immune checkpoint inhibitors targeting PD-1 and PD-L1, as well as other mAbs.

Second, mAbs can function by targeting tumor-associated antigens (TAAs) on cancer cells for enhanced NK cell cytotoxicity through ADCC [102]. Several clinical trials are investigating mAbs targeting CD20 in combination with CD19-directed CAR NK cells, for the treatment of B cell lymphomas (NCT05618925, NCT05379647, NCT03579927, among others). CD20 is highly expressed on B cells and also restricted to the B cell lineage, thus representing a secondary target with high on-target specificity. CD38 is also being explored as a target for mAbs in combination therapy for the treatment of multiple myeloma in combination with BCMA-directed CAR NK cells with CD38 KO to avoid fratricide (NCT05182073). Other mAbs being explored in combination with CAR NK cell therapies include cetuximab (anti-EGFR), for the treatment of head and neck cancer (NCT06239220) and bevacizumab (anti-VEGF-A) for the treatment of glioblastoma (NCT06061809).

### Cytokines

Another strategy used to enhance the functional response of CAR NK cells is combined administration of cytokines. Unlike T cells, NK cells cannot produce their own IL-2 and depend on external survival signals for sustained effector functions. Administration of exogenous cytokines, such as IL-2 and IL-15, can support the proliferation and cytotoxicity of infused NK cells in vivo [103–105]. Several clinical trials are investigating administration of IL-2 in combination with CAR NK cells (NCT03415100, NCT05336409, NCT05395052, NCT01974479, NCT06255028). IL-2 is generally given as a single subcutaneous injection simultaneously with CAR NK cells, with one suspended study (NCT01974479) exploring alternate-day dosing for 6 doses starting a day before CAR NK cell infusion. Subcutaneous IL-2 allows for slower absorption and longer persistence than intravenous administration, reducing the need for high doses that could cause severe side effects, such as capillary leak syndrome, hypotension, and organ failure [106, 107].

Another cytokine being explored in combination with CAR NK cell therapy is IL-15. IL-15 is a critical cytokine in NK cell development and for maintaining effector functions [108, 109]. Administration of IL-15 may also induce fewer adverse events compared with IL-2 [110]. All clinical trials currently employing IL-15 in combination with CAR NK cells, are using the proprietary IL-15 agonist N-803, manufactured and licensed by ImmunityBio, Inc. (NCT03228667, NCT05618925, NCT04847466, NCT04390399, NCT06239220, NCT06334991). N-803 consists of a mutated IL-15 (N72D) bound to an IL-15 receptor α/IgG1 Fc fusion protein. The N72D variant of IL-15 has an increased affinity for the IL-15Rβγ complex, resulting in a four–fivefold increase in biological activity compared to the native molecule [111]. Additionally, by complexing human IL-15 directly to an IL-15 receptor α/IgG1 fusion protein, the in vivo half-life is drastically increased to ~ 20 h compared with ~ 1 h for native IL-15 in mice [112].

### Other molecules

In addition to mAbs and cytokines, other combination strategies are being actively explored to augment the biological activity of infused CAR NK cells. One clinical trial, NCT05410717, is currently investigating the use of nicotinamide adenine dinucleotide (NAD) or Cannabidiol (CBD) in combination with CAR NK cells in patients with advanced solid cancers. Cannabidiol may enhance NK cell functions, including cytotoxicity, migration, and proliferation, however, the mechanisms behind these effects are not fully elucidated [113, 114]. Cannabidiol in itself, is also speculated to be cytotoxic against tumors and induce lymphopenia of activated B cells and T cells, while NK cells are less sensitive to its cytotoxic effects [115–117]. NAD may exert similar enhancements in the biological activity of NK cells, by enhancing proliferation, cytotoxicity and cytokine production [118].

## Results of clinical trials with CAR NK cells

In this section, results obtained from clinical trials involving CAR NK cells are described. Out of the 120 registered clinical trials, we have identified clinical data from a total of 16 distinct studies focused on the clinical application of CAR NK cells. These studies provide valuable insights into the safety, efficacy, and potential of CAR NK cell therapies across a range of medical conditions. The current results of the studies are summarized in Table 4 and introduced further below.

**Table 4:** Clinical trials of CAR NK cell agents

Target antigen	Program	Trial ID	Phase	Indication	Cell source	Number of patients (efficacy evaluable)	Dose levels	mPLT (range)	Safety	Efficacy outcome	Publication
CD19	CAR19/IL-15	NCT03056339	Phase I/II	Indolent lymphoma, CLL, DLBCL, MCL, B-ALL, Lymphoplasmacytic lymphoma	CB-NK	41 (37)	100k/kg, 1M/kg, 10M/kg 800M	4 (2–10)	No DLT, no GvHD, no ICANS, 1G1 CRS	Day 30 and 100 ORR: 48.6% Day 30 and 10 CR rate: 27.0% and 29.7% 1 year PFS: 32% 1 year OS: 68%	[119]
	NKX019	NCT05020678	Phase I	B-Lymphoma, B-ALL, CLL, WM	PB-NK	19 (14) Recent update: 26	300M, 1.0B, 1.5B	4 (2–10)	No DLT, no GvHD, no ICANS, 2G1 CRS, 2G2 CRS, 1G3 CRS	ORR = 10/14 (CR = 8, PR = 2) Recent update: ORR = 19/26	[122], [123]
	FT596	NCT05934097	Phase I	B-Lymphoma	iPSC	20 (17)	30M, 90M, 300M	4	No DLT, no GvHD, no ICANS, 1G1 CRS, 1G2 CRS	ORR = 5/8 (Monotherapy)	[125]
										ORR = 4/9 (Combination)	
	CNTY-101	NCT05336409	Phase I	B-Lymphoma	iPSC	12 (10)	100M, 300M, 900M	4 (2–5)	No DLTs or GVHD 2G1 CRS, 2G2 CRS, 1G1 ICANS	ORR = 4/10 (CR = 3, PR = 1)	[126]
CD33	NA	NCT05008575	Phase I	AML	NA	10 (7)	600M, 1.2B, 1.8B	5 (3–8)	7G1 CRS, 1G2 CRS	ORR = 6/10 (MRD-CR = 6)	[127]
	NA	NCT02944162	Phase I	AML	NK-92	3 (3)	300M, 800M, 1.0B, 1.5B, 3.0B, 5.0B	NA	2G1 CRS	ORR = 2/3	[128]
NKG2DL	NKX101	NCT04623944	Phase I	AML	PB-NK	6 (6) Recent update: 20	100M, 300M, 1.0B, 1.5B	2 (1–3)	No DLT, no GvHD, no ICANS, No CRS No disc. due to AE	ORR = 4/6 (CR/CRi = 4) Recent update: ORR = 5/20	[129], [130], [131], [132]
	NA	NCT03415100	Phase I	Colorectal cancer	PB-NK	3 (3)	Multiple	4 (4–4)	No DLT, no GvHD, no ICANS, No CRS	All SD, with one patient with CMR pr. PET	[133]
PD-L1	PD-L1 t-haNK	NCT04390399	Phase II	Pancreatic Cancer	NK-92	63	2.0B	NA	Treatment related (TR) SAE’s were uncommon (6%), no TR deaths were reported	mOS in 3rd line subjects (n = 34) was 6.2 months. OS for ITT population (N = 78) of 3rd, 4th and 5th line was 5.8 months	[136]
	PD-L1 t-haNK	NCT04050709	Phase I	Solid tumors	NK-92	9 (9)	2.0B, 4.0B	NA	No DLT, no GvHD, no ICANS, no CRS	4 SD. Additionally, a CR was reported on a compassionate expanded use in a patient with metastatic pancreatic cancer	[134] [135]
	CCCR-NK92	NCT03656705	Phase II*	NSCLC	NK-92	1 (1) (Case report)	10M, 20M, 50M, 100M	NA	One severe case of CRS was reported	The patient achieved SD	[138]
BCMA	FT576	NCT05182073	Phase I	MM	iPSC	9 (9)	100M, 300M	5 (3–10)	No DLT, no GvHD, no ICANS, no CRS No disc. due to AE	ORR = 3/7 (MR = 1, PR = 1, vgPR = 1)	[139]
PSMA	ALF501	NCT03692663	Phase I	CRPC	NA	1 (1) (Case report)	500M	NA	No liver and kidney toxicity was observed	SD, with PSA reduction of 97.5% from baseline	[140]
ROBO1	NA	NCT03941457	Phase I	Pancreatic Cancer	NK-92	1 (1) (Case report)	1.0B	NA	Moderate fever that quickly resolved	The patient achieved SD	[142]
MUC1	NA	NCT02839954	Phase I	Solid tumors	NK-92	13 (10)	1.0B	NA	Severe cytokine storm and/or bone marrow suppression were not observed	9 SD	[143]
HER2	HER2 t-haNK	NCT03383978	Phase I	Glioblastoma	NK-92	9 (9)	10M, 30M, 100M	NA	No DLT, no GvHD, no ICANS, no CRS	Median OS in the whole cohort was 31 weeks. Two patients treated with the highest dose level were alive 116 and 97 weeks after therapy	[92]

*OS* Overall Survival, *PFS* Progression Free Survival, *ORR* Objective Response Rate, *CR* Complete Response, *PR* Partial Response, *SD* Stable Disease, *DLT* Dose Limiting Toxicity, *CRS* Cytokine Release Syndrome, *ICANS* Immune effector Cell-Associated Neurotoxicity Syndrome, *ITT* Intention To Treat, *AE* Adverse Events, *GvHD* Graft versus Host Disease, *mPLT* median Prior Lines of Therapy, *NA* Not Applicable, *CMR* Complete Metabolic Response, *PET* Positron Emission Tomography, *PSA* Prostate Specific Antigen, *MR* Minimal Response, *vgPR* very good Partial Response

*This study is designated as a Phase I study in the clinical trial entry NCT03656705, however, designated a Phase II study in the original paper

### Results against CD19-expressing malignancies

#### M.D. Andersson cancer center (NCT03056339)

A recently completed, first-in-human phase 1/2 clinical trial, investigated the use of CD19-directed CAR NK cells for the treatment of patients with relapsed or refractory (R/R) B cell malignancies (NCT03056339). In the trial, 37 patients received CAR NK cells that were engineered to express an anti-CD19 CAR, secrete IL-15 and harbor an iCasp9 safety switch. The NK cells were sourced from a different CB donor for each patient [119]. In the dose escalation part of the study, 11 patients received escalating doses of CAR NK cells of either 100 thousands cells/kg, 1 million cells/kg, or 10 millions cells/kg. In the dose expansion part, 11 patients received doses of 10 million cells/kg, while 15 patients received a flat dose of 800 million CAR NK cells. No dose limiting toxicities (DLTs) were encountered and no patients developed GvHD or ICANS. Only one patient developed CRS (grade 1). The study showed promising efficacy with a 1-year overall survival (OS) rate of 68% and a progression free survival (PFS) of 32%. The overall response (OR) rates after 30 and 100 days were both 48.6%, with 37.8% of patients achieving complete response (CR) after 1 year.

In a post hoc analysis, the authors identified CB characteristics as an important predictor of outcome, with time from collection to cryopreservation and nucleated red blood cell (NRBC) count being significant independent predictors. Patients that received CAR NK cells from CB units with ≤ 24 h from collection to cryopreservation and containing ≤ 8 × 10^7^ NRBC’s, had an improved 1-year PFS (hazard ratio (HR) = 0.094) and OS (HR = 0.073) compared to remaining patients.

Notably, the authors additionally identified trogocytosis of CD19 as a significant predictor of relapse. Patients with high levels of trogocytosis showed a worse 1-year OS of 38.5% versus 82.6%, and a CR rate of 7.7% versus 56.5%, compared to patients with low levels of trogocytosis. Trogocytosis may negatively impact the efficiency of CAR NK cells by lowering tumor antigen density, thereby decreasing sensitivity of tumor cells to CAR NK cells, and by triggering self-recognition and fratricidal killing of CAR NK cells expressing the trogocytosed antigen [120].

#### Nkarta, Inc. (NCT05020678)

Another CD19-directed CAR NK, NKX019, is being tested in a currently recruiting clinical trial to treat CD19 + B cell malignancies (NCT05020678). NKX019 is a cryopreserved PB derived allogeneic CAR NK cell product that incorporates a CD19-directed CAR that uses OX40 as its costimulatory signaling domain and uses mbIL-15 for enhanced in vivo persistence. Preclinical data has demonstrated NKX019’s ability to rapidly clear CD19 + cells, both in vitro and in vivo [121]. In the clinical trial, 19 patients were included in an initial assessment, including 14 with B cell lymphomas and five with leukemias, all of whom had a median of four prior lines of treatment. These patients received NKX019 at varying doses, including 300 million, 1 billion, and 1.5 billion cells. Patients received lymphodepleting preconditioning with Cy/Flu prior to treatment. At the time of cut-off, no DLTs were observed, and there were no instances of CRS, ICANS, or GvHD. Efficacy was demonstrated across various histologies, with eight patients achieving CR, including five who achieved CR after a single treatment cycle. Additionally, three patients with NHL experienced deepening of response from partial response (PR) to CR with additional treatment cycles. In the highest dose cohorts, there was an 80% objective response rate (ORR) and a 70% CR rate in patients with NHL. Notably, five patients maintained CR for over six months, including one LBCL patient who sustained CR for more than nine months [122]. In a recent data update, 26 participants were included, showing an ORR of 73%. Interestingly, the update featured data from four patients who had received retreatment after initial CR and subsequent PD. After just one cycle of re-dosing, all four patients achieved CR again, with two remaining in CR, highlighting the potential efficacy of re-dosing in this context [123].

#### Fate Therapeutics, Inc. (NCT04245722)

FT596 is a CAR NK cell therapy targeting CD19, which was evaluated in a clinical trial that has since been terminated by the sponsor (NCT04245722). FT596 is derived from an engineered iPSC cell line, and incorporates multiple different synthetic edits. Apart from the knock-in of the CD19 directed CAR, it also harbors a hnCD16 and mbIL-15. In preclinical studies, it has shown efficient tumor cell clearance, especially when combined with an anti-CD20 mAb [124]. In this clinical trial, FT596 was assessed for the treatment of R/R B cell lymphomas. Two distinct treatment regimens were explored: Regimen A, featuring FT596 as monotherapy, and Regimen B, where FT596 was combined with rituximab or obinutuzumab, two anti-CD20 mAbs. The lymphodepleting regimen Cy/Flu preceded single doses of FT596, administered at levels ranging from 30 million cells to 900 million cells during dose escalation. Preliminary data was reported for 20 patients, 10 treated in each regimen. Patients had received a median of four prior lines of therapy and seven of the 20 patients had received prior CAR-T cell therapy. No DLTs or cases of ICANS or GvHD were observed, and only two instances of CRS were reported, graded as 1 and 2, respectively. Among the 17 efficacy-evaluable patients, five out of eight in Regimen A and four out of nine in Regimen B achieved an objective response following the first FT596 treatment cycle. Notably, at single-dose levels of ≥ 90 million cells, eight out of 11 efficacy-evaluable patients achieved an objective response, with seven of them experiencing CR. Moreover, among the four patients with prior CAR T-cell therapy treated at ≥ 90 million cells, two achieved CR [125]. The sponsor of the study, Fate Therapeutics, Inc., decided to discontinue FT596 in favor of a next-generation CD19-targeting CAR NK product, FT522, which is undergoing testing in NCT05950334.

#### Century Therapeutics, Inc. (NCT05336409)

Another CAR NK therapy CNTY-101, targeting CD19, is currently under investigation in a clinical trial, currently recruiting, to treat R/R B cell lymphoma (NCT05336409). The cells are derived from iPSC clones and feature six synthetic modifications: KO of HLA-I and HLA-II, and knock-in of a CD19-CAR with an FMC63 scFv, an EGFR-based safety switch, HLA-E to prevent host NK cell rejection and a gene for secretable IL-15. The trial has so far tested doses of 100 million, 300 million and 1 billion cells. Patients receive Cy/Flu conditioning, along with low-dose IL-2 during treatment. Preliminary data from the trial include 12 patients, with 10 of them being eligible for efficacy evaluation, who had previously undergone a median of four lines of therapy, including three patients with prior CD19-directed CAR-T treatment. The treatment was well-tolerated at these doses, with four cases of CRS, graded 1 and 2 and a single case of grade 1 ICANS. No DLTs, or GvHD were observed. Notably, the study found no evidence of allo-rejection, likely due to the allo-evasion strategies employed. Overall, the efficacy outcomes so far have been mixed, with an ORR of 40%. At the two lowest doses of CNTY-101, two patients achieved CR, one achieved PR, and four experienced disease progression. Among the CRs, one was notably durable lasting 10 months at the lowest dose of 100 million cells. The other CR occurred in a patient who had undergone five prior lines of therapy, including previous CD19 CAR-T therapy. In a separate dosing schedule with three weekly infusions, one patient achieved stable disease (SD) with 300 million cells. At the highest dose, one patient progressed immediately, while another achieved CR [126].

### Results against CD33-expressing malignancies

#### Xinqiao Hospital of Chongqing (NCT05008575)

In a clinical trial, CAR NK cells were engineered to target CD33 for the treatment of R/R AML (NCT05008575). As of the latest trial update in 2022, the recruitment status remains unknown. Efficacy assessments have been completed for 10 patients, all of whom had undergone a median of five prior lines of treatment. The study explored three distinct dose groups, which followed a lymphodepletion regimen comprising Cy/Flu. In the first dose group, three patients received three rounds of CAR NK cells, comprising 600 million to 1.8 billion cells, with a 7-day interval between each dose. In the second dose group, three patients received a single dose of 1.8 billion CAR NK cells. The third dose group included four patients who received three rounds of 1.8 billion CAR NK cells with a 7-day interval. Seven patients experienced grade 1 CRS, while one patient experienced a grade 2 CRS event. Impressively, six out of the ten patients achieved minimal residual disease-negative complete remission (MRD-CR) at the day 28 assessment. As of the data cutoff point, two of these six patients remained in an ongoing response, with one of them having exceeded the 5-month milestone [127].

#### PersonGen BioTherapeutics Co., Ltd. (NCT02944162)

Another trial investigating the use of CD33-targeted CAR NK cells was conducted to treat AML (NCT02944162). As of the latest trial update in 2016, the recruitment status remains unknown. These CAR NK cells were derived from a NK-92 cell line and featured a lentiviral construct incorporating both CD28 and 4-1BB costimulatory domains. Preliminary outcomes were reported for three patients, each with distinct results. The first patient received CAR NK cell doses ranging from 300 million cells to 1 billion cells. During the treatment course, the patient experienced grade 1 CRS. While the patient initially responded, with a bone marrow evaluation revealing 0% blasts and an MRD level of 1.7 × 10^−3^, relapse eventually occurred. The second patient received three doses of CAR NK cells, comprising 300 million, 600 million and 1 billion cells. This patient experienced a grade 1 CRS and initially responded to treatment, however, relapsed after one month with bone marrow examination showing 75% blasts, with 49% CD33 + expression. The third patient received three doses of CAR NK cells, comprising 1 billion cells, 3 billion cells and 5 billion cells cells. Regrettably, this patient exhibited no response to the treatment. These cases underscore the varying responses observed in individuals treated with CD33-targeted CAR NK cells, highlighting the complexity of this therapeutic approach and the need for further investigation [128].

### Results against NKG2DL-expressing malignancies

#### Nkarta, Inc. (NCT04623944)

NKG2D ligands represent an alternative targeting modality for CAR based therapy. To explore these targets, a clinical trial was conducted examining the CAR NK product NKX101 in patients with R/R AML (NCT04623944). This clinical trial is currently active, while not recruiting. NKX101 is an allogeneic CAR NK product derived from PB and designed for cryopreservation. It incorporates mbIL-15 for enhanced in vivo persistence and integrates OX40 as a costimulatory domain within the CAR construct. The clinical trial comprised two distinct approaches: In one cohort, patients underwent prior conditioning with Cy/Flu and in the other cohort, the conditioning regimen consisted of Cy/Flu combined with cytarabine (Cy/Flu/Ara). The Cy/Flu cohort comprised 30 patients, having received a median of two prior lines of therapy. The patients received doses ranging from 100 million to 1.5 billion cells, administered at varying dosing frequencies. NKX101 was found to be well tolerated, with only 12% of patients having grade 1 and 2 infusion reactions, 12% of patients having grade 1 and 2 CRS, a single case of grade 2 ICANS and no GvHD or DLTs. Among the 30 dosed patients, five achieved CR or complete response with incomplete hematologic recovery (CRi). Furthermore, in the highest doses of 1 billion or 1.5 billion cells, four out of 18 patients achieved a CR or CRi [129]. Turning to the Cy/Flu/Ara cohort, the conditioning regimen was hypothesized to induce the upregulation of NKG2D ligands on the tumor cells. Patients in this cohort had a median of two prior lines of therapy. In an initial data update, six patients had undergone dosing, each receiving 1.5 billion cells. Notebly, there were no reported instances of CRS, ICANS, or GvHD. Furthermore, four out of six patients within this cohort achieved a CR or CRi [130, 131]. However, in a recent update, 14 more patients had been treated, with only one responder achieving CR, leading to the sponsor deciding to discontinue further development of the therapy and stop recruiting [132].

#### The Third Affiliated Hospital of Guangzhou Medical University (NCT03415100)

In another trial, NKG2D ligands again served as the target for CAR NK cells (NCT03415100). As of the latest trial update in 2018, the recruitment status remains unknown. In this trial, the extracellular domain of the human NKG2D receptor was fused with the cytoplasmic signaling domain of DAP12, an adaptor molecule associated with activating NK cell receptors, and the construct was electroporated into NK cells. Three patients with metastatic colorectal cancer (mCRC) received local infusions of these CAR NK cells, with one patient receiving autologous CAR NK cells, while two patients received allogeneic CAR NK cells from haploidentical family donors. NK cell expansion was facilitated using K562 feeder cells expressing mbIL-15, 4-1BBL, and mbIL-21. There were no instances of ICANS, GvHD, DLTs or serious adverse events among the patients and only mild grade 1 CRS was observed. One patient received two infusions of 20 million and 100 million CAR NK cells, prepared with autologous NK cells. Another patient was treated with four infusions of CAR NK cells, ranging from 100 to 700 million cells, prepared with allogeneic haploidentical NK cells. Both patients exhibited SD in peritoneal target lesions as determined by CT scanning. A third patient, who presented with unresectable mCRC that had progressed after three lines of prior treatment, underwent a treatment regimen involving six infusions of allogeneic haploidentical CAR NK cells. This regimen included administering 500 million CAR NK cells twice a week for the first week, 1 billion CAR NK cells twice a week in the second week, and 2 billion CAR NK cells twice a week in the third week. A notable reduction in tumor size in the liver region was observed shortly after the second injection [133].

### Results against PD-L1-expressing malignancies

#### ImmunityBio, Inc. (NCT04050709 and NCT04390399)

CAR-modified NK-92 cells are also being tested in targeting PD-L1, with PD-L1 t-haNK being in the forefront, currently being the subject of multiple active clinical trials (Table 1). This particular therapeutic approach incorporates endoplasmic reticulum retained IL-2 and a high-affinity variant of CD16. In a phase I clinical trial, which is currently active but not recruiting, focused on locally advanced or metastatic solid cancers, PD-L1 t-haNK showed early potential (NCT04050709). Two distinct cohorts received treatments involving either 2 billion cells or 4 billion cells twice a week. PD-L1 t-haNK was safe and tolerable, with no instances of CRS, ICANS, or GvHD and most reported adverse events were classified as grade 1 or 2. Among the nine patients enrolled, four achieved SD [134]. Additionally, a compassionate use expanded access investigational new drug (IND) reported a CR in a patient with metastatic pancreatic cancer, according to a company press release [135]. Furthermore, the same therapeutic modality was subjected to testing in a larger phase II study, also currently active but not recruiting, targeting locally advanced or metastatic pancreatic cancer (NCT04390399). PD-L1 t-haNK was administered at doses of 2 billion cells in combination with aldoxorubicin and N-803, an IL-15 superagonist, in a cohort of 83 patients. The median OS stood at 5.7 months, with 26 out of 83 patients still alive at the data cut-off. The median PFS was 2.3 months with 37% of the subjects achieving SD for a duration exceeding 8 weeks. In the subgroup analysis, the median OS for those in the 3rd line of treatment (N = 38) reached 6.3 months, while in the 4th line or greater (N = 40), it reached 5.0 months [136].

#### Xinxiang Medical University (NCT03656705)

In another completed phase I trial, researchers investigated a CAR NK cell product named CCCR-NK92 for the treatment of non-small cell lung cancer (NSCLC) (NCT03656705). Unlike traditional CARs, this therapy, which is derived from an NK-92 cell line, features a unique chimeric costimulatory converting receptor (CCCR). Instead of the typical scFv, CCCR-NK92 incorporates the extracellular domain of PD-1 to interact with PD-L1. Additionally, it encompasses the hinge region, transmembrane, and cytoplasmic domains of NKG2D, along with the cytoplasmic domain of 4-1BB to facilitate co-stimulatory signals. This design aims to transform the inhibitory signal from PD-1 into an activating signal within the TME. In preclinical studies, CCCR-NK92 was shown to exhibit enhanced anti-tumor activity against PD-L1 positive H1299 cells, compared to un-transduced cells [137]. A case report from the conducted trial was released in 2022. The patient received doses of CCCR-NK92 twice weekly, starting at 10 million CCCR-NK92 cells and escalating to 100 million cells per injection. While the patient achieved SD, she also experienced a severe case of CRS. Interestingly, this CRS event, while atypical for CAR NK cells, led the authors to speculate that it may be attributed to the unique CCCR-NK92 design, potentially resulting in overstimulation of NK cells and subsequent overproduction of cytokines, such as IFN-γ and tumor necrosis factor [138].

### Results against BCMA-expressing malignancies

#### Fate Therapeutics, Inc. (NCT05182073)

BCMA is another target that has been pursued in clinical trials with CAR NK cells and yielded clinical data (NCT05182073). FT576, which is developed from an engineered clonal iPSC master cell line, integrates four functional enhancements: a CAR targeting BCMA, hnCD16 to boost cytotoxicity, mbIL-15 to enhance persistence, and CD38 KO to provide resistance against mAb-mediated fratricide. Data was reported for nine patients in a phase 1 study, although it has now been terminated, with two dosage levels of 100 million cells and 300 million cells being assessed. The study involved patients who were heavily pretreated with a median of five previous lines of therapy. There were no instances of CRS, ICANS, GvHD, DLTs or serious adverse events among the patients. Among the nine patients evaluated for efficacy, three experienced a reduction in myeloma disease burden (ranging from 38 to 97%), with two demonstrating a confirmed OR [139].

### Results against PSMA-expressing malignancies

#### Allife Medical Science and Technology Co., Ltd. (NCT03692663)

Another study investigates the efficacy and safety of PSMA targeting CAR NK cells for treating CRPC (NCT03692663). As of the latest trial update in 2022, the recruitment status remains unknown. Patients received infusions of CAR NK at an interval of 3-months, for a total of three courses, with each infusion comprising 500 million cells. Preliminary data is currently available for a single patient, for which the safety profile was reported to be encouraging, with no liver or kidney toxicity during the course of treatment. The PFS for this patient was 12 months at the time of data-cutoff, at which point the patient’s prostate-specific antigen (PSA) levels had decreased by 97.5% from baseline levels [140].

### Results against ROBO1-expressing malignancies

#### Asclepius Technology Company Group Co., Ltd. (NCT03941457)

In another clinical trial, researchers investigated the novel target, ROBO1, for the treatment of pancreatic cancer (NCT03941457). As of the latest trial update in 2019, the recruitment status remains unknown. Prior to clinical study start, the therapy demonstrated moderate efficacy in vitro, however, in mouse models, they were unable to demonstrate extended survival benefits [141]. Derived from the NK-92 cell line, these CAR NK cells were genetically modified using lentiviral vectors with a CAR comprising a ROBO1-specific scFv, a CD8-derived transmembrane domain, a CD3ζ signaling domain, and a 4-1BB costimulatory domain. Additionally, an iCasp construct was integrated into the therapeutic design. A case report from the clinical study, published in 2020, highlighted the treatment of a patient with unresectable PDAC and liver metastases using ROBO1 CAR NK cells. The patient received a dose of 1 billion ROBO1-specific CAR NK cells via percutaneous injections targeting the liver metastases and intravenous injections for PDAC. The therapy presented as well-tolerated, with no instances of CRS and no other significant adverse events reported. Although the treatment controlled the pancreatic lesion and liver metastasis within five months, the patient, unfortunately, passed away due to disease progression, resulting in an OS time of 8 months [142].

### Results against MUC1-expressing malignancies

#### PersonGen BioTherapeutics Co., Ltd. (NCT02839954)

MUC1 has emerged as another promising target in cancer immunotherapy. In a phase 1/2 clinical trial, anti-MUC1 CAR NK cells were derived from a NK-92 cell line to treat lung, pancreatic, colon, and ovarian cancers, with a specific focus on tumors co-expressing PD-L1 and MUC1 (NCT02839954). As of the latest trial update in 2016, the recruitment status remains unknown. A lentiviral vector was utilized to transduce cells with the anti-MUC1 CAR, incorporating CD28 and 4-1BB as costimulatory domains. Additionally, the design included a truncated PD-1 peptide to redirect inhibitory signals. Each participant received a treatment dose of 1 billion cells. Of the 13 patients initially enrolled, three withdrew for reasons not specified. Among the remaining 10 patients, nine patients achieved SD as their best response, while one patient experienced disease progression. Importantly, no cases of severe CRS or significant bone marrow suppression were reported [143].

### Results against HER2-expressing malignancies

#### Johann Wolfgang Goethe University Hospital (NCT03383978)

In a first-in-human clinical trial, currently active but not recruiting, researchers are pioneering the use of CAR NK cells in the treatment of glioblastoma, with a specific focus on HER2-positive glioblastoma (NCT03383978). The CAR NK cells utilized in this study were derived from the NK-92 cell line and engineered with a HER2-specific scFv, a CD8α hinge region, a CD28 costimulatory domain and a CD3ζ signaling domain. Prior to initiating the clinical trial, this therapy demonstrated encouraging efficacy in preclinical studies through selective cytotoxicity and serial-killing capabilities [93, 144]. For the clinical trial, GMP-compliant cell products were generated from a single clone of lentivirally transduced NK-92 cells. Nine patients with recurrent HER2-positive glioblastoma received single doses of irradiated CAR NK cells intracranially at doses ranging from 10 million cells to 100 million cells during relapse surgery. Importantly, the therapy exhibited excellent tolerability, with no DLTs, CRS or ICANS. Results revealed that five patients displayed SD, while four patients experienced disease progression. The median PFS for all patients was 7 weeks, with a range of 2 to 37 weeks, and the median OS for the entire cohort was 31 weeks, ranging from 18 to 135 weeks. As the next phase of investigation, the researchers intend to explore an expansion cohort, implementing repetitive local injections of CAR NK cells [92].

### Other case reports

A recent case of sudden death was reported following administration of CAR NK cells for the treatment of lung cancer [145]. The patient, in their 50 s, was diagnosed with left upper lung adenocarcinoma, T2N2M1, stage IV and had undergone resection and received two chemotherapy perfusions one year prior to CAR NK cell infusion. The patient received a total of four infusions of CB-derived CAR NK cells and developed fever after each injection. Following the fourth round of CAR NK cell infusion, the patient developed severe CRS, presented as fever, chills, vomiting, incontinence, dyspnea, and loss of consciousness. The patient’s heart rate finally dropped and resuscitation was unsuccessful leading to death of the patient. Blood analysis showed elevated concentrations of proinflammatory cytokines, as well as increased numbers of NK cells and total lymphocytes. Post-mortem analysis of the patients tissues, revealed significant damage to heart, lung, brain, and kidney, and was associated with increased numbers of NK cells in the tissues.

Another case of death following CAR NK cell therapy was reported on a patient diagnosed with R/R DLBCL [146]. The 59-year-old male patient initially received chemotherapy as well as anti-CD19 CAR T cell therapy, but relapsed with heart metastasis after 12 months. The patient then received chemotherapy followed by a CAR NK cell infusion of 5.6 million cells/kg, which resulted in grade 2 CRS. The patient experienced symptomatic relief and the researchers found a reduction in the cardiac mass. The patient then received another round of chemotherapy followed by haploidentical allogeneic hematopoietic stem cell transplantation (allo-SCT). The researchers reported SD following HSCT, however, the patient experienced severe fungal pneumonia which later resulted in septic shock and death of the patient.

A third case study describes a 24-year-old male patient diagnosed with T-ALL, who developed central nervous system relapse following treatment with chemotherapy and allo-SCT [147]. The patient received an intrathecal injection of CB-derived anti-CD7 CAR NK cells. The patient experienced an initial worsening of neurological symptoms and had elevated levels of proinflammatory cytokines in the cerebrospinal fluid, which the researchers linked to CRS induced by the CAR NK cells. Despite initial worsening, the patient experienced improvements in symptoms and his bone marrow and PB remained in CR with complete donor chimerism more than nine months after CAR NK cell infusion.

## Conclusion and future perspectives

CAR NK cell-based therapies represent a promising new advancement in cancer immunotherapy, offering important advantages over the widely successful CAR T cell therapy. However, despite a rapid increase in registered clinical trials investigating CAR NK cells, there is still a lack of comprehensive analysis on their safety and efficacy as well as the manufacturing strategies and treatment protocols currently under investigation. This review gathered data from 120 registered clinical trials on CAR NK cells and examined the preliminary outcomes published from 16 of these trials.

Recent efforts in CAR NK cell therapy development have focused on enhancing efficacy, persistence, and safety across a range of applications, including hematologic and solid cancers, autoimmune diseases, and infectious conditions. Key strategies include optimizing the choice of NK cell sources (PB-NK, CB-NK, iPSC-NK, or NK-92) and manufacturing platform, each offering unique benefits for scalability and potency. Additionally, genetic modifications represent a central approach for enhancing CAR NK cell function, including cytokine engineering (e.g., IL-15 and IL-7) to improve in vivo survival, and engineered CD16 to boost ADCC. Other approaches aim to circumvent immune challenges, such as allo-evasion techniques to reduce immune rejection and safety switches (e.g., iCasp9) for controlled cell elimination in case of severe adverse effects. Bispecific CAR designs and combination therapies with monoclonal antibodies further represent strategies aiming to amplify CAR NK cell activity, particularly in solid cancers where the immunosuppressive TME poses additional barriers. Through these multifaceted efforts, CAR NK cell therapies are being refined to maximize therapeutic potential and broaden their clinical applications.

Outcomes from recent clinical trials have consistently demonstrated a favorable safety profile of CAR NK cells, when compared to CAR T cell therapies. Across the broad range of included studies, CAR NK cell treatments have largely avoided the severe toxicities commonly associated with CAR T cells, such as ICANS and high-grade CRS. Remarkably, there have been no reports of GvHD, despite the majority of studies utilizing NK cells derived from allogeneic cell sources and doses reaching as high as 5 billion CAR NK cells. While mild CRS (grade 1–2) has been observed in several studies, and one case involving sudden death following CAR NK cell infusion, the general absence of severe adverse events indicate a high feasibility of using allogeneic CAR NK cells as a safe treatment option.

The most robust clinical responses so far have been reported in hematologic cancers, particularly B-cell malignancies. High response rates and CRs have been achieved with CAR NK cell therapies targeting CD19, often even in patients who previously failed CAR T cell therapy. Additionally, other targets, such as CD33 for AML and BCMA for MM, are being explored with intriguing preliminary results, although larger studies are needed to support these findings. Although some trials report durable responses lasting several months, many trials have reported rapid relapse following initial responses, emphasizing the need for improved strategies to enhance the persistence and efficacy of CAR NK cells.

CAR NK cells are also being explored in solid cancers, including glioblastoma (HER2-t-haNK) and pancreatic cancer (PD-L1 t-haNK). Whereas the tolerance remained high in these studies, the efficacy was generally lower compared to hematologic cancers. Solid cancers pose unique challenges such as an immunosuppressive TME and physical barriers to NK cell infiltration [26]. Despite these challenges, some studies have reported disease stabilization and partial responses, suggesting potential clinical utility for CAR NK cells in these contexts.

The flexibility to develop CAR NK cell therapies from diverse starting materials and manufacturing strategies may play a significant role in influencing treatment outcomes. For instance, CB-derived CAR NK cells, especially those cryopreserved within 24 h of collection, were associated with superior response rates and OS in a recently completed phase 1/2 trial. These insights suggest that optimizing the manufacturing and source of CAR NK cells may serve as important milestones in order to further enhance their clinical performance. Dosing may also play a more significant role for CAR NK cells than CAR T cells due to their limited persistence and expansion post-infusion. The relatively short lived CAR NK cells may exert their effects more immediately following infusion, thus requiring higher doses to achieve similar effects. Appropriately, the most promising responses have been observed in trials using infusions of above 90 million cells, whereas CAR T cells are typically administered in the range 50–100 million cells. Repeated dosing of CAR NK cells has also shown promise, with one trial demonstrating that patients who relapsed after an initial CR, regained CR status after a single re-dosing. The high tolerability and off-the-shelf potential of CAR NK cells enable flexible dosing and multiple infusions without severe side effects, making it a versatile treatment option.

In light of emerging results, we identify three key areas requiring innovation to advance the field of CAR NK cell therapy.

**Overcoming the TME.** Solid cancers pose significant challenges for CAR NK cells due to their immunosuppressive environment and physical barriers that limit cell infiltration and activity. Enhancing CAR NK cell efficacy in these contexts requires innovative approaches such as combining CAR NK cells with immune checkpoint inhibitors to mitigate suppression or introducing genetic modifications that improve NK cell fitness and resilience to TME-induced dysfunction. Additionally, engineering CAR NK cells to secrete chemokines or enzymes that facilitate tissue penetration could address physical barriers in the TME.

**Enhancing persistence.** The inherently short-lived nature of NK cells, while minimizing long-term toxicity risks, restricts therapeutic durability. Strategies to extend in vivo persistence include engineering survival-promoting genes (e.g., anti-apoptotic factors), implementing allo-evasion mechanisms to reduce host-versus-graft immune responses, or utilizing controlled cytokine delivery systems to sustain NK cell proliferation and activity. These innovations will likely be critical for achieving sustained therapeutic responses, particularly in relapsed or refractory cancers.

**Optimizing manufacturing processes.** The ability to derive CAR NK cells from various sources (e.g., PB, CB, iPSC, or NK-92) enables scalable, off-the-shelf therapeutic production. However, clinical-grade CAR NK cell manufacturing is complex, with variability in cell product characteristics potentially impacting outcomes. To ensure consistent quality, further optimization of donor or cell source selection, cell culture conditions, cryopreservation protocols, and dosing strategies is needed. Incorporating automated, standardized manufacturing platforms and robust quality control measures will be vital to producing high-quality, cost-effective infusion products at scale.

CAR NK cells are also being evaluated for the treatment of autoimmune disorders, comprising over a third of registered clinical trials in 2024 so far. Off-the-shelf CAR NK cells may offer a safer and more cost-effective alternative to CAR T cells in this regard, which is especially beneficial when used to treat non-life threatening diseases. In addition, similar long-term persistence of CAR cells may not be required to achieve a reset of the immune system, offering a potential benefit of using short-lived CAR NK cells, compared to CAR T cells that may persist for years.

In conclusion, while CAR NK cells are still in the early stages of clinical development, their excellent safety profile, promising efficacy in hematologic malignancies, and the potential for broad applicability in different cancers, and recently also autoimmune disorders, highlight their promise as a next-generation immunotherapy. A particularly promising avenue in this field is the development of allogeneic off-the-shelf CAR NK cell therapies, which offer the advantages of scalability, cost-effectiveness, and immediate availability compared to autologous approaches. Further research and innovation will be key to overcoming current limitations and unlocking their full potential.

## Supplementary Information

Additional file 1

## Data Availability

Data is provided within the manuscript or supplementary information files.

## Abbreviations

ADCC: Antibody dependent cell cytotoxicity
ADR: Alloimmune defence receptor
AE: Adverse Events
ALL: Acute lymphocytic leukemia
allo-SCT: Allogeneic hematopoietic stem cell transplantation
AML: Acute myeloid leukemia
BPDCN: Blastic plasmacytoid dendritic cell neoplasm
CAR: Chimeric antigen receptor
CBD: Cannabidiol
CB: Cord blood
CCCR: Chimeric costimulatory converting receptor
CLL: Chronic lymphocytic leukemia
CMR: Complete Metabolic Response
CR: Complete response
CRi: Complete response with incomplete hematologic recovery
CRS: Cytokine release syndrome
Cy/Flu: Cyclophosphamide and flurdarabine regimen
DCs: Dendritic cells
DLBCL: Diffuse large B cell lymphoma
DLT: Dose limiting toxicity
EMA: European Medicines Agency
ER: Endoplasmic reticulum
FDA: U.S. Food and Drug Administration
FL: Follicular lymphoma
GMP: Good manufacturing practices
GM-CSF: Granulocyte–macrophage colony-stimulating factor
GvHD: Graft-versus-host disease
HLA: Human leukocyte antigen
hESC: Human embryonic stem cells
hnCD16: High affinity, non-cleavable CD16
iCasp9: Inducible caspase-9
ICANS: Immune effector cell-associated neurotoxicity syndrome
IL: Interleukin
IL-15RF: IL-15/IL-15 receptor alpha fusion protein
IND: Investigational new drug
iPSC: Induced pluripotent stem cell
ITP: Primary immune thrombocytopenia
ITT: Intention To Treat
KIR: Killer immunoglobulin-like receptor
KO: Knock-out
MCL: Mantle cell lymphoma
MDS: Myelodysplastic syndromes
MDSC: Myeloid-derived suppressor cells
MM: Multiple myeloma
MR: Minimal Response
MRD-CR: Minimal residual disease-negative complete remission
mAbs: Monoclonal antibodies
mbIL-15: Membrane-bound IL-15
mbIL-21: Membrane-bound IL-21
mCRPC: Metastatic castration-resistant prostate cancer
mPLT: Median prior lines of therapy
MZL: Marginal zone lymphoma
NAD: Nicotinamide adenine dinucleotide
NK: Natural killer
NRBC: Nucleated red blood cell
ORR: Objective response rate
OS: Overall survival
PB: Peripheral blood
PDAC: Pancreatic ductal adenocarcinoma
PET: Positron Emission Tomography
PFS: Progression free survival
PR: Partial response
PSA: Prostate Specific Antigen
PSMA: Prostate-specific membrane antigen
R/R: Relapsed or refractory
SLEDAI: SLE remission and the maintenance of a Disease Activity Index
scFv: Single-chain variable fragment
SD: Stable disease
TAAs: Tumor associated antigens
tEGFR: Truncated epidermal growth factor receptor
TME: Tumor microenvironment
vgPR: very good Partial Response
WM: Waldenström's macroglobulinemia
